# Multi-omics analysis reveals the pathogenesis of db/db mice diabetic kidney disease and the treatment mechanisms of multi-bioactive compounds combination from *Salvia miltiorrhiza*

**DOI:** 10.3389/fphar.2022.987668

**Published:** 2022-09-29

**Authors:** Zhuo Xu, Xiang Xiang, Shulan Su, Yue Zhu, Hui Yan, Sheng Guo, Jianming Guo, Er-Xin Shang, Dawei Qian, Jin-ao Duan

**Affiliations:** ^1^ Jiangsu Collaborative Innovation Center of Chinese Medicinal Resources Industrialization, National and Local Collaborative Engineering Center of Chinese Medicinal Resources Industrialization and Formulae Innovative Medicine, Jiangsu Key Laboratory for High Technology Research of TCM Formulae, Nanjing University of Chinese Medicine, Nanjing, China; ^2^ Shanghai Institute of Materia Medica, Chinese Academy of Sciences, CAS, Shanghai, China

**Keywords:** multi-bioactive compounds combination, diabetic kidney disease, multi-omics, Salvia miltiorrhiza, PI3K/Akt/FoxO signaling pathways

## Abstract

Diabetic kidney disease (DKD) is a common diabetic complication. *Salvia miltiorrhiza* has significant therapeutic effects on diabetes complications, although the mechanism remains unclear. Here, biochemical indicators and pathological changes were used to screen out the optimal *Salvia miltiorrhiza* multi-bioactive compounds combination. Metabolomics, transcriptomics and proteomics were used to explore the pathogenesis of DKD. RT-PCR and parallel reaction monitoring targeted quantitative proteome analysis were utilized to investigate treatment mechanisms of the optimal *Salvia miltiorrhiza* multi-bioactive compounds combination. The db/db mice showed biochemical abnormalities and renal lesions. The possible metabolic pathways were steroid hormone biosynthesis and sphingolipid metabolism. The 727 differential genes found in transcriptomics were associated with biochemical indicators via gene network to finally screen 11 differential genes, which were mainly key genes of TGF-β/Smad and PI3K/Akt/FoxO signaling pathways. *Salvia miltiorrhiza* multi-bioactive compounds combination could significantly regulate the Egr1, Pik3r3 and Col1a1 genes. 11 differentially expressed proteins involved in the two pathways were selected, of which 9 were significantly altered in db/db mice compared to db/m mice. *Salvia miltiorrhiza* multi-bioactive compounds combination could callback Q9DBM2, S4R1W1, Q91Y97, P47738, A8DUK4, and A2ARV4. In summary, *Salvia miltiorrhiza* multi-bioactive compounds combination may ameliorate kidney injury in diabetes through regulation of TGF-β/Smad and PI3K/Akt/FoxO signaling pathways.

## Introduction

Diabetic kidney disease (DKD) is one of the most common microvascular complications in diabetic patients, which is also the main cause of end-stage kidney disease (ESKD) (Chapman et al., 2016). Recently, the pooled data from 54 countries reveal that more than 80% of ESKD cases are caused by diabetes, hypertension or a combination of both (United States Renal Data System, 2012). The pathogenesis of DKD is dynamic and complex, which involves interplays of a variety of factors, such as glucose metabolism disorder, kidney hemodynamic changes, abnormal expression of various cytokines, oxidative stress, kidney tissue inflammation and heredity (Mauer et al., 1984; Forbes and Cooper, 2013; Fakhruddin et al., 2017). At present, clinic treatments for DKD mainly rely on the control of blood glucose and lipid, with applications of angiotensin-converting enzyme inhibitor or angiotensin receptor antagonist medicine (Lv et al., 2015; Sharma et al., 2017). However, the therapeutic effect is unsatisfactory and the occurrence and development of DKD cannot be effectively regressed.

DanShen is derived from the root of *Salvia miltiorrhiza* (Xiang et al., 2019). Emerging evidence suggests that *Salvia miltiorrhiza* can be used as a potential adjunctive drug in the treatment of diabetic microangiopathy including diabetic retinopathy and DKD (Zequn et al., 2021). The main bioactive components of *Salvia miltiorrhiza* include water-soluble phenolic acids and fat-soluble tanshinones (Xiang et al., 2019). Previously, we determined the main components in the stem and leaves of *Salvia miltiorrhiza*, mostly containing salvianolic acid B, rosmarinic acid, and other water-soluble salvianolic acid components. Importantly, we found that the extract of root, stem and leaves of *Salvia miltiorrhiza* and their total phenolic acid components showed protective effects on diabetic kidney damage and gastrointestinal damage (Gu et al., 2017; Cai et al., 2018; Xiang et al., 2019). However, the molecular mechanism of salvianolic acid and tanshinone has not been investigated.

Recently, metabolomics is increasingly used as a technical means to discover biomarkers in epidemiology, which can capture metabolic changes and identify biomarkers of the disease procession (Connor et al., 2010; Mao et al., 2017). Transcriptomics utilizes high-throughput sequencing technology to comprehensively determine almost all transcripts in organs or tissues, and is mainly used for screening differentially expressed genes, searching for new functional genes, exploring the relationship between disease and gene expression, and molecular diagnosis of disease (Papadopoulos et al., 2017; Li Z. et al., 2019)**.** Further, quantitative proteomics based on tandem quality markers and LC-MS/MS has been widely used for the detection of proteins in cells and tissues with high sensitivity (Chen et al., 2017).

Here, we sought to explore the pathogenesis of DKD via multi-omics approaches, including metabolomics, transcriptomics, and proteomics, and clarify the effects mechanism of *Salvia miltiorrhiza* bioactive compounds.

## Materials and methods

### Animal experiments

All the experimental procedures and protocols used in this study were reviewed and approved by the Institutional Animal Ethics Committee of Nanjing University of Chinese Medicine (Nanjing, China), Animal license number: SCXK (su) 2015–0001. Seven-week-old male congenital gene-deficient db/db mice and age-matched wild-type db/m littermates were purchased from the Animal Model Research Center of Nanjing University. Before and throughout experimentation, the animals were housed in a specific pathogen-free barrier facility with constant humidity (ca. 60% ± 2%) and temperature (ca. 22 ± 2°C), and with a light/dark cycle of 12 h. Mice had unrestricted access to water and chow.

In this biological activity experiment, five bioactive compounds were combined with a ratio of stem-leaf and root, including salvianolic acid B, rosmarinic acid, lithospermic acid, Danshensu, and tanshinone ⅡA. Multi-bioactive compounds combination compatibility proportion was shown in Supplementary Table S1. As shown in Supplementary Table S2, the db/db mice were randomly divided into different groups after the adaptation period of 2 weeks.

Body weight and fasting blood glucose levels were monitored weekly. Blood glucose levels were measured from tail vein using a One Touch Ultra II blood glucose monitoring system (Life Scan). After drug administration, mice fasted in the metabolic cages for 12 h urinary collection. At the end of the study, mice were sacrificed under anesthesia, and blood was collected for biochemical parameters and metabolomics study. The blood samples were centrifuged at 3,000 rpm for 10 min. Then the serum samples were separated and stored at -80°C. The right kidney was removed and fixed with 10% neutral-buffered formalin for pathological analysis. The kidney cortex was isolated and frozen in liquid nitrogen (Dai et al., 2018).

### Biochemical indicators measurements and pathological analysis

The therapeutic efficacy of db/db mice was evaluated for the levels of FBG (fasting blood glucose), INS (insulin), TC (total cholesterol), TG (triglycerides), Scr (Serum creatinine), and BUN (blood urea nitrogen) in serum. Part of the kidney cortex was removed for hematoxylin eosin (HE) and periodic acid-schiff (PAS) staining to observe pathological changes in kidney tissue, degrees of fibrosis tissue hyperplasia, and structures of glomeruli and tubules through electron microscope (× 200).

### Metabolomics study on serum and urine samples (Dai et al., 2018)

Serum samples were extracted with three times the volume of acetonitrile, and urine samples were extracted with one time the volume of acetonitrile to precipitate proteins. The mixture was vortexed for 1.5 min and centrifuged at 13,000 rpm for 15 min 2 µL of the supernatant was injected into the UPLC-QTOF/MS and analyzed in positive and negative modes. For other details see Supplementary Materials.

### Transcriptomics study on kidney tissues

RNAseq was performed to identify differentially expressed genes between the control and diabetic mice. Total RNA was extracted using the mirVana miRNA Isolation Kit (Ambion) following the manufacturer’s protocol. RNA integrity was evaluated using the Agilent 2100 Bioanalyzer (Agilent Technologies). The samples with RNA Integrity Number ≥7 were subjected to the subsequent analysis. The libraries were constructed using TruSeq Stranded mRNA LTSample Prep Kit (Illumina) according to the manufacturer’s instructions. These libraries were sequenced on the Illumina sequencing platform (HiSeqTM 2500 or Illumina HiSeq × Ten) and 125 bp/150 bp paired-end reads were generated. Raw data (raw reads) were processed using the Trimmomatic (version 0.36) software (Bolger et al., 2014). In this step, clean data (clean reads) were obtained by removing reads containing adapter and ploy-N or low quality reads that percentage of bases with Q_phred_ ≥ 20 were more than 50%. Volume and concentration of libraries and quality of raw sequence reads (Supplementary Table S3) can be seen in Supplementary Materials.

### Real-time fluorescence quantitative PCR

Total RNA was extracted from kidney tissue samples using mirVana^TM^ RNA Isolation Kit according to the manufacturer’s specifications (Ambion). The yield of RNA was determined using a NanoDrop 2000 spectrophotometer (ThermoFisher), and the integrity was evaluated using agarose gel electrophoresis stained with ethidium bromide. Quantification was performed with a two-step reaction process: reverse transcription and real-time PCR. Real-time fluorescence quantitative PCR was performed with a two-step reaction process: reverse transcription (RT) and PCR. Each RT reaction has two steps. The first step was 0.5 μg RNA, 2 μL of 4×gDNA wiper Mix, add Nuclease-free H_2_O to 8 μL. Reactions were performed in a GeneAmp^®^ PCR System 9700 (Applied Biosystems, United States) for 2 min at 42°C. The second step: 2 μL of 5 × HiScript II Q RT SuperMix (cat. no. R223-01, Vazyme) was added to gDNA-removed reaction. Reactions were performed in a GeneAmp^®^ PCR System 9700 (Applied Biosystems, United States) for 15 min at 50°C, 5 s at 85°C. The 10 μL RT reaction mix was then diluted×10 in nuclease-free water and held at -20°C. Real-time PCR was performed using LightCycler^®^ 480 Ⅱ Real-time PCR Instrument (Roche, Swiss) with 10 μL PCR reaction mixture that included 1 μL of cDNA, 5 μL of 2×ChamQ SYBR qPCR Master Mix (cat. no. Q311-02, Vazyme), 0.2 μL of forward primer, 0.2 μL of reverse primer and 3.6 μL of nuclease-free water. Reactions were incubated in a 384-well optical plate (Roche, Swiss) at 95°C for 30 s, followed by 40 cycles of 95°C for 10 s, 60°C for 30 s. Each sample was run in triplicate for analysis. At the end of the PCR cycles, melting curve analysis was performed to validate the specific generation of the expected PCR product. The expression levels of mRNAs were normalized to Gapdh and were calculated using the 2^-△△Ct^ method (Livak and Schmittgen, 2001). Data was analyzed using student’s t test by Microsoft Excel. The primer sequences were designed based on the mRNA sequences obtained from the NCBI database as Supplementary Table S4.

### Proteomics study on kidney tissues

Kidney tissue samples were ground and pulverized in liquid nitrogen and transferred into low protein binding tubes and lysed with 300 µL lysis buffer supplemented with 1 mM PMSF. Then, samples were further lysed with sonication on ice. The parameters were set as 1s/1s intervals, 3 min, and 80 W of power. After sonication, samples were centrifuged at 12,000 g for 10 min at room temperature to remove insoluble particles that were repeated once to further exclude precipitation. Protein concentration was determined by Bradford assay and the samples were aliquoted and stored at −80°C. Total proteins (15 µg) of each sample were acquired and separated by 12% SDS-PAGE gel. All samples were then trypsinized and labeled. The labeling peptide solutions were lyophilized and stored at -80°C. Protein separation was performed on an 1100 HPLC System (Agilent) using an Agilent Zorbax Extend RP column (5 μm, 150 mm × 2.1 mm). Tryptic peptides were separated at a flow rate of 300 μL/min and monitored at 210 and 280 nm. Dried samples were harvested from 8 min to 50 min and elution buffer was collected every minute and numbered from 1 to 10 with the pipeline. The separated peptides were lyophilized for MS detection. The MS/MS data were analyzed for protein identification and quantification using Proteome Discoverer^TM^ 2.2 (ThermoFisher Crop.). The local false discovery rate was estimated with the integrated PSPEP tool in the ProteinPilot Software to be 1.0% after searching against a decoy concatenated uniport *Mus musculus* protein database. Other protein separations and the separated peptides detection details see Supplementary Materials.

### PRM targeted quantitative proteome analysis

The sample mix was fractionated on an Agilent 1100 liquid chromatograph at pH 10. A total of six fractions were collected and run in DDA mode to obtain the protein lists, which were used to set up a scheduled PRM assay. The DDA raw files were searched against database in which the Biognosys iRT peptide sequences were added with ProteomeDiscover (version 2.3). Trypsin was used as the digestion enzyme. Search criteria included carbamidomethylation of cysteine as a fixed modification and oxidation of methionine and acetyl (protein N terminus) as variable modifications. Up to two missed cleavages were allowed. The mass tolerance for the precursor was 10 ppm and 0.02 Da for MS/MS, respectively. Identifications were filtered to obtain FDR of 1% at the peptide and the protein levels. A list of peptides from DDA analysis was prepared for PRM validation. Samples were loaded onto a precolumn (100 μm × 3 cm, C_18_, 3 μm, 150 Å) and separated on an analytical column (75 μm × 15 cm, C18, 3 μm, 120 Å) at a flow rate of 300 nL/min (mobile phases A: 2% acetonitrile, 0.1% formic acid; mobile phases B: 95% acetonitrile, 0.1% formic acid). For other details see Supplementary Materials.

### Statistical analysis

SPSS 16.0 software (SPSS Inc.) was used for statistical analysis. Statistical results were expressed as the mean ± standard deviation. Comparisons between groups were made using one-way ANOVA, followed by Tukey’s multiple comparison test. *p*-value < 0.05 was considered as significant.

## Results

### Biochemical indicators and histopathological changes

After 2 weeks of adaptive feeding, the average fasting blood glucose level (fasting 8–12 h) of db/db mice was approximately 11.1 mmolL ^−1^, which can be considered diabetic (Li K. et al., 2019). They were then randomly divided into administration groups (Supplementary Table S1).

**FIGURE 1:**
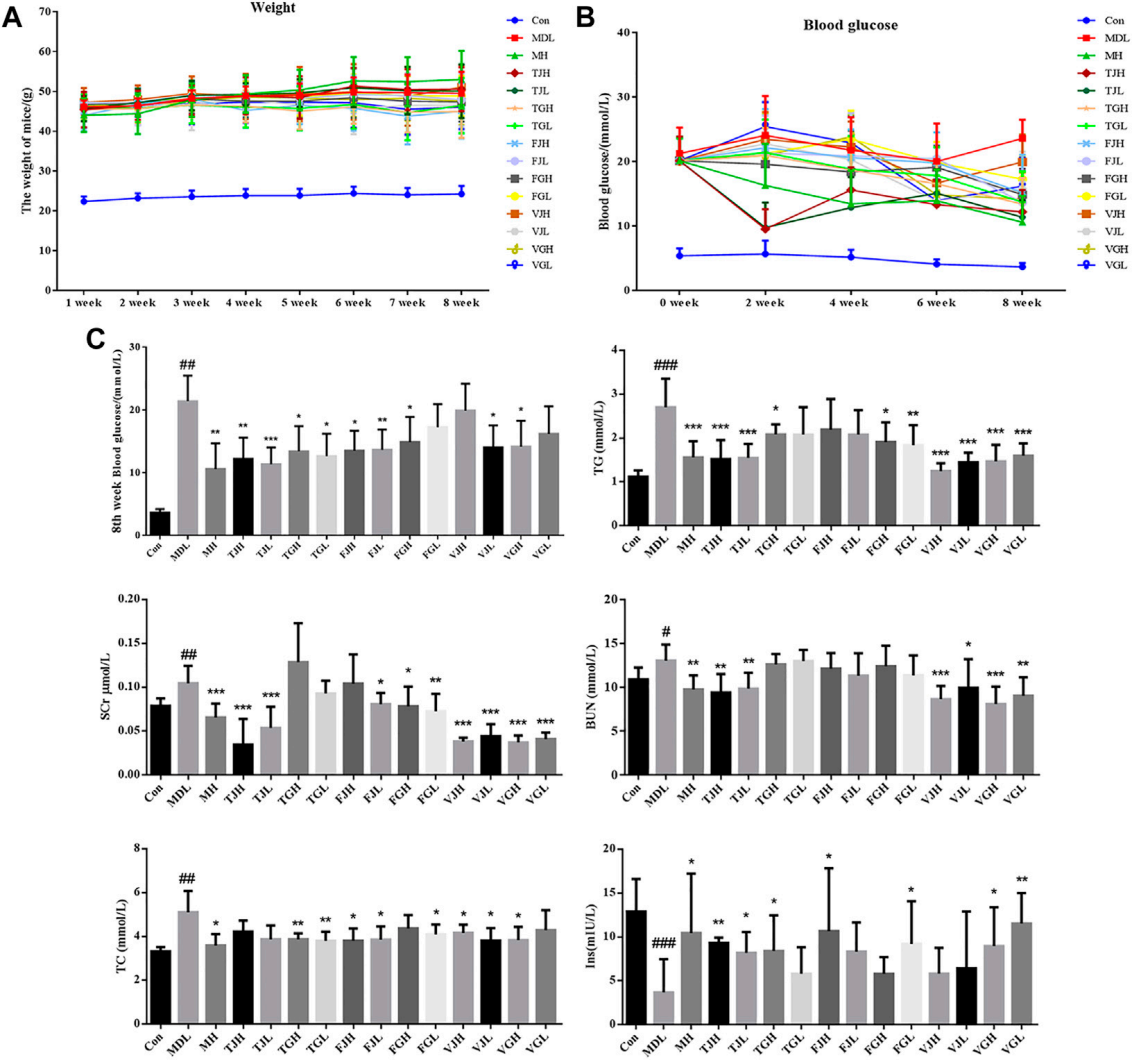
**(A)**. Multi-week weight change curve (*n* = 10); **(B)**. Blood glucose change curve (*n* = 10); **(C)**. Determination of biochemical indicators among control group, model group and administration groups; (^#^ *p* < 0.05; ^##^ *p* < 0.01; ^###^ *p* < 0.001: models vs. control; ^*^ *p* < 0.05; ^**^ *p* < 0.01; ^***^ *p* < 0.001: treatment groups vs. models).

During 8 weeks of treatment of multiple bioactive compounds from *Salvia miltiorrhiza*, body weight and blood glucose levels were recorded **(** Figures 1A,B). The levels of FBG, TC, TG, BUN, and Scr in serum of db/db model group were increased significantly compared with that of the control group (Figure 1C), which indicates that the mouse model had significant kidney lesions. After the preventive treatment, the blood glucose in TJH, TJL, TGH, TGL, FJH, FJL, FGH, VJL, and VGH group showed a trend of decreasing compared with the disease group. The levels of multiple biochemical indicators showed different degrees of a callback trend, especially VGH group, which had a significant regulating effect on all six indicators detected. TJH and TJL can significantly reduce BUN and Scr, which are important indicators to evaluate the degree of kidney injury. At the same time, TJH and TJL have significant up-regulated effects on Ins, which can be used to evaluate the diagnosis and classification of diabetes (Figure 1C).

**FIGURE 2:**
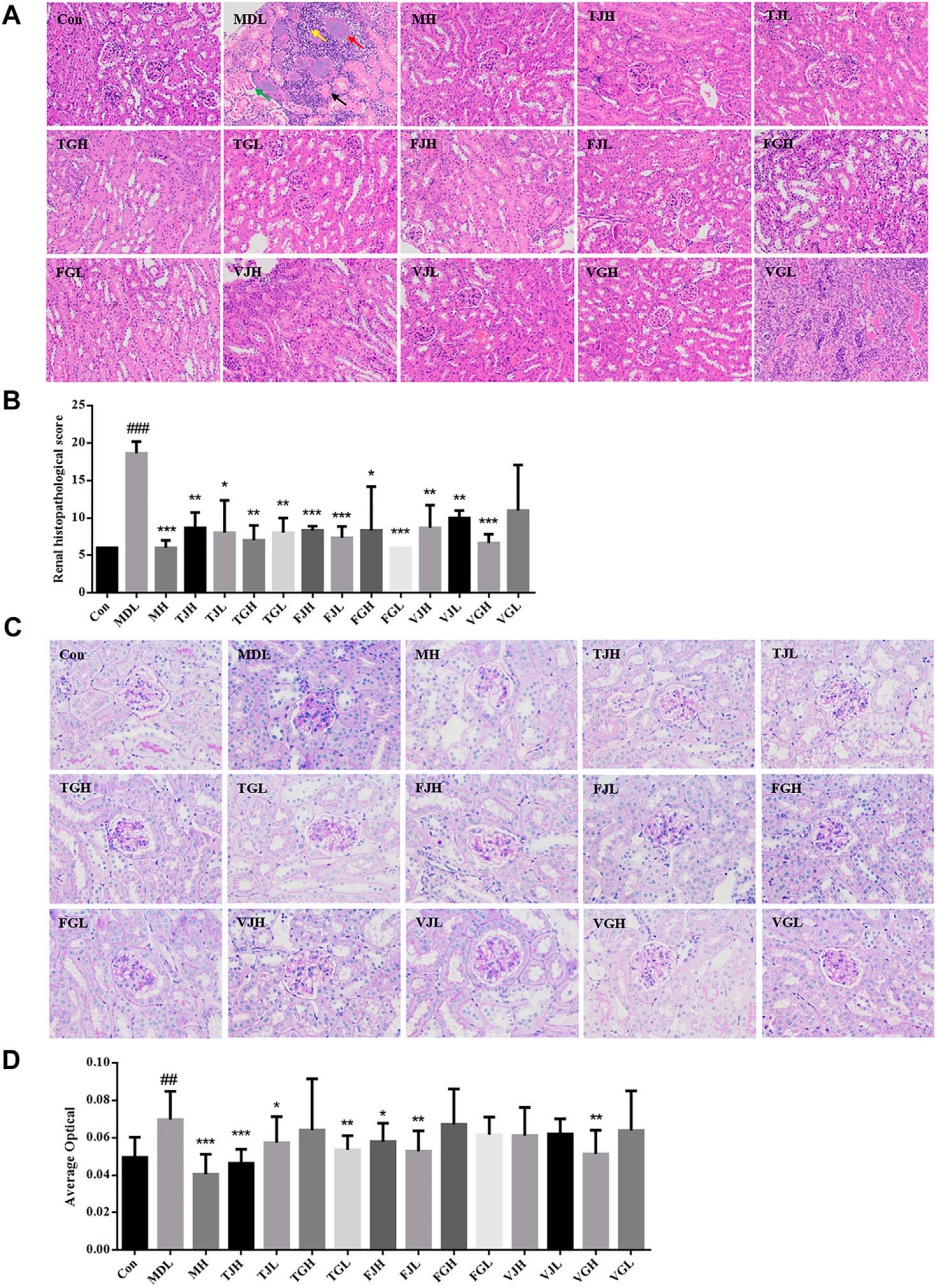
**(A)**. Pathological section of kidney tissue revealed by HE staining (×200); **(B).** Pathological section score; **(C)**. Pathological section of kidney tissue revealed by PAS staining (×400); **(D)**. Average optical (^#^ *p* < 0.05; ^##^ *p* < 0.01; ^###^ *p* < 0.001: models vs control; ^*^ *p* < 0.05; ^**^ *p* < 0.01; ^***^ *p* < 0.001: treatment groups vs models) (Black arrow: the cell nucleus was deep-stained or fragmented and dissolved. Red arrow: necrosis, structural disorder in the glomerular cells. Green arrow: a small amount of kidney tubular necrosis calcification. Yellow arrow: inflammatory cells in the tubulointerstitial).

As shown in Figure 2A, the results of HE were consistent with the biochemical analysis. The kidney lesions in the db/db model group were more severe and the pathological scores were significantly higher than those in the control group (*p* < 0.001). The kidney tissue was extensively necrotic and the structure was disordered. Also, the kidney tubular structure disappeared and the kidney tubular epithelial cells were necrotic. The cell nucleus was deep-stained or fragmented and dissolved, as shown by the black arrow in Figure 2A; necrosis, structural disorder in the glomerular cells, as shown by the red arrow; a small amount of kidney tubular necrosis calcification can be observed, as shown by the green arrow; many inflammatory cells can be seen in the tubulointerstitial, as indicated by the yellow arrow. According to the results of pathological scoring (Figure 2B), after multi-bioactive compounds combination administration, except for VGL groups, the extent of lesions in the other groups was significantly different from that in the disease group. Fewer areas of tissue necrosis were observed in VGL group and little areas of inflammatory cells infiltrated could be seen in TJH, TJL, TGL, FJL, VJH, and VJL groups. The histomorphology of TGH, FGH, FGL, and VGH groups was nearly restored to the control group.

PAS staining can reveal the thickening degree of glomerular basement membrane, which has been widely used in the diagnosis and research of diabetes (Fu and Campbell-Thompson, 2017). The results of PAS staining showed that compared with the control group, the glomerular area was increased, the basement membrane was thickened, and the average optical density value was elevated significantly in the db/db group (*p* < 0.01). (Figures 2C,D). Compared with the disease group, the average optical values of TJL group had the most significant downregulation effect, and the results of TGL, FJL and VGH groups were particularly similar to those of the blank group.

### Optimal ratio screening

Cluster analysis was performed based on the results of biochemical indicators and pathological section results (Supplementary Figure S1). The results showed that both the db/db model group and the control group were separately classified into a single category, indicating that the two groups could be significantly distinguished. TJH and TJL groups can be classified into one class with the positive drug group. VGH group can be classified into one class with positive drug group in the secondary level. Initially, we determined the optimal ratio was three monomers combined with ratio of stem-leaf (TJ) and five monomers combined with ratio of root (VG).

### Metabolomics alternations

**FIGURE 3:**
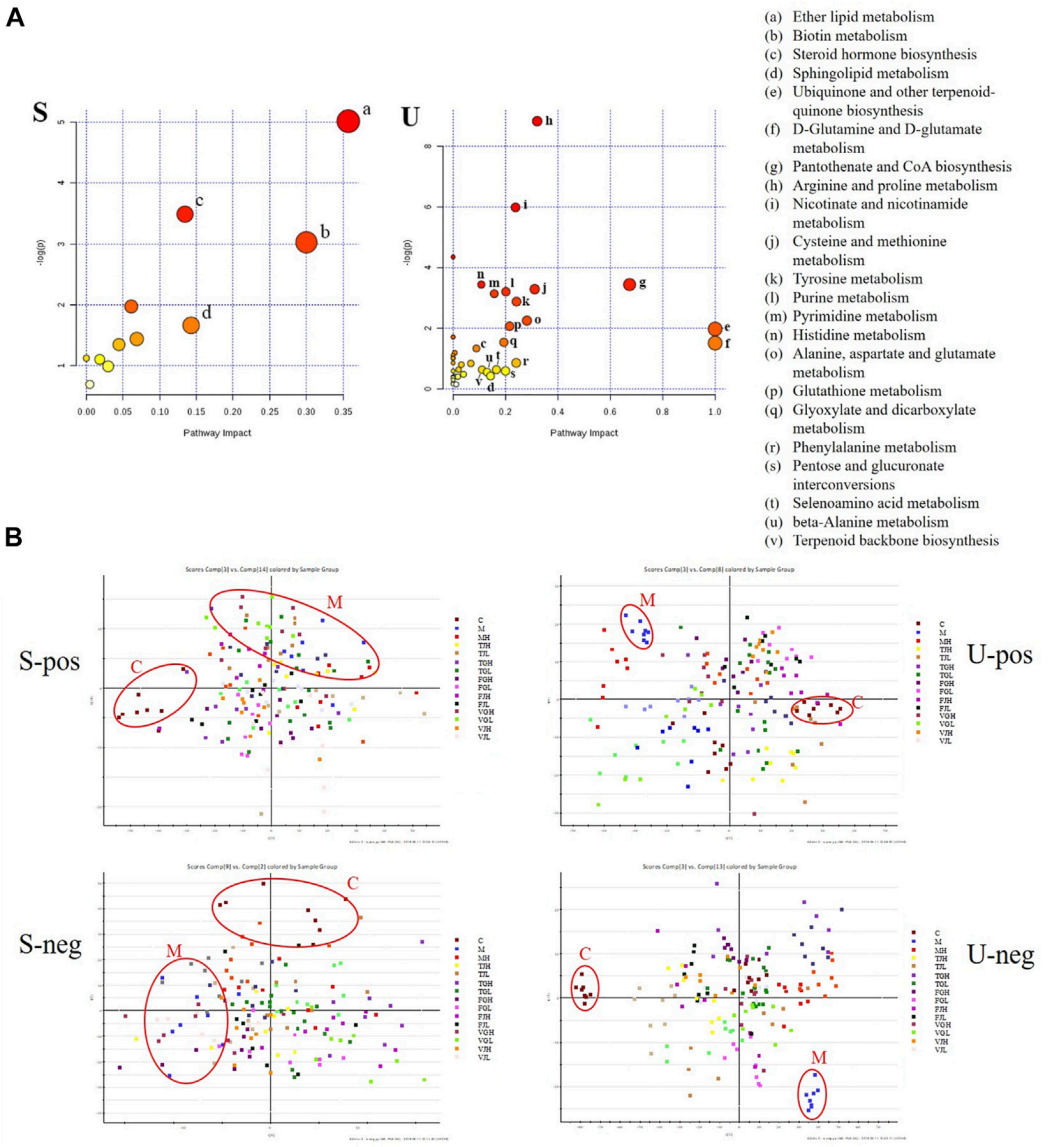
**(A)**. Summary of pathway analysis with MetPA of potential metabolites in serum (S) and urine (U); **(B)**. PLS-DA scores plots for serum (S) and urine (U) samples from models, controls and treatment group in positive and negative ion mode.

The data of serum and urine metabolic profiles of control and model mice were patterned by orthogonal partial least squares discriminant analysis (Supplementary Figure S2), which indicates that metabolic abnormalities occurred in db/db mice compared with control group. Potential markers were chosen based on their contribution to the variation and correlation of the data set of VIP-plot. The differential metabolites, generally the metabolites with VIP >1 are considered as differential metabolites. The *t*-test (student’s t test) was used to verify whether the differences in metabolites between groups were significant. A total of 14 endogenous metabolites in serum and 113 metabolites in urine samples were identified in db/db mice compared with the control group (Supplementary Table S5). These potential metabolites were imported into MetPA (https://www.metaboanalyst.ca/) and KEGG database to access related metabolic pathways. Serum samples metabolic pathway value larger including ether lipid metabolism and biotin metabolism; urine samples metabolic pathway value larger including ubiquinone and other terpenoid-quinone biosynthesis, D-glutamine and D-glutamate metabolism, and pantothenate and CoA biosynthesis. Steroid hormone biosynthesis and sphingolipid metabolism are common pathways involved in both serum and urine samples (Figure 3A). As revealed by the PLS-DA score plots for serum and urine samples from each group shown (Figure 3B), after treatment with multi-bioactive compounds combination from *Salvia miltiorrhiza*, the animals’ abnormal metabolic profile was improved and the affected metabolites in TJ and VG groups tended to return to normal levels.

### Transcriptomics alternations

**FIGURE 4:**
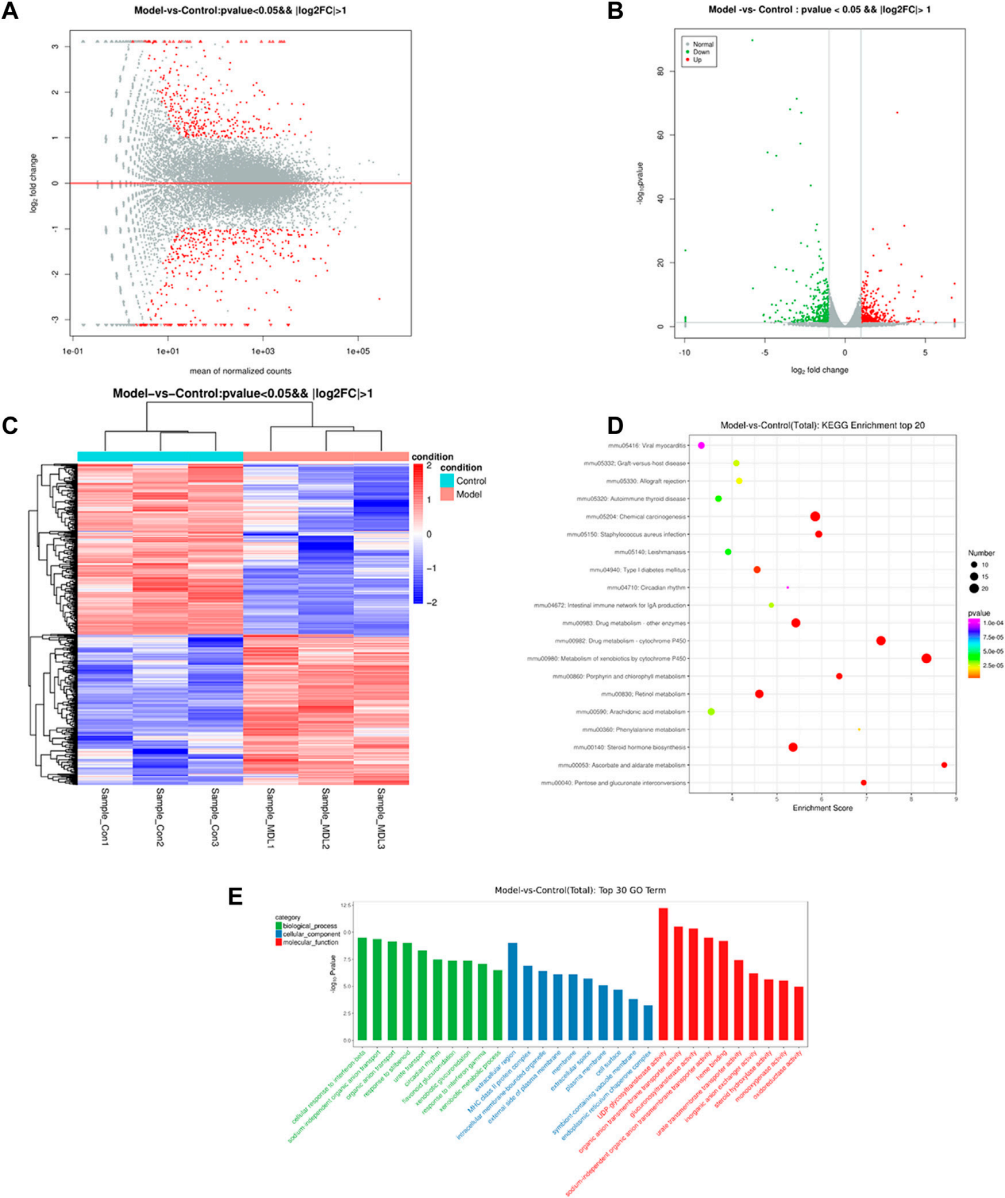
The differentially expressed genes in the control and model groups of MA map **(A)**, volcano map **(B)** and heat map **(C)**. Analysis result of GO **(D)** between the control and model groups. KEGG enrichment **(E)** of the control and model groups.

The results of sequencing data quality preprocess can be seen in Supplementary Table S6. A total of 727 differentially expressed genes were found, including 340 up-regulated genes and 387 down-regulated genes. Further analyses revealed distinct gene clusters between disease and control groups (Figures 4A,B). This was corroborated by unsupervised gene-level clustering, revealing distinctive patterns of significantly upregulated and downregulated genes when comparing the db/db model groups and control groups (Figure 4C). After the differentially expressed genes were obtained, gene ontology (GO) enrichment analysis was performed to describe their functions from the three levels of biological process (BP), cellular component (CC) and molecular function (MF) (Figure 4D). Molecular functional level analysis showed diabetic pathway-related processes, including UDP-glycosyltransferase activity, glucuronosyl-transferase activity, steroid hydroxylase activity, monooxygenase activity, and oxidoreductase activity. We also conducted pathway enrichment analysis of differentially expressed genes by KEGG database (combined with KEGG annotation results) and calculated the significance of pathway enrichment of differentially expressed genes in each pathway entry by hypergeometric distribution test (Figure 4E). The results suggest the involvement of diabetic pathway-related, including arachidonic acid metabolism, phenylalanine metabolism, steroid hormone biosynthesis, and pentose and glucuronate interconversions.

### Weighted gene co-expression network analysis

**FIGURE 5:**
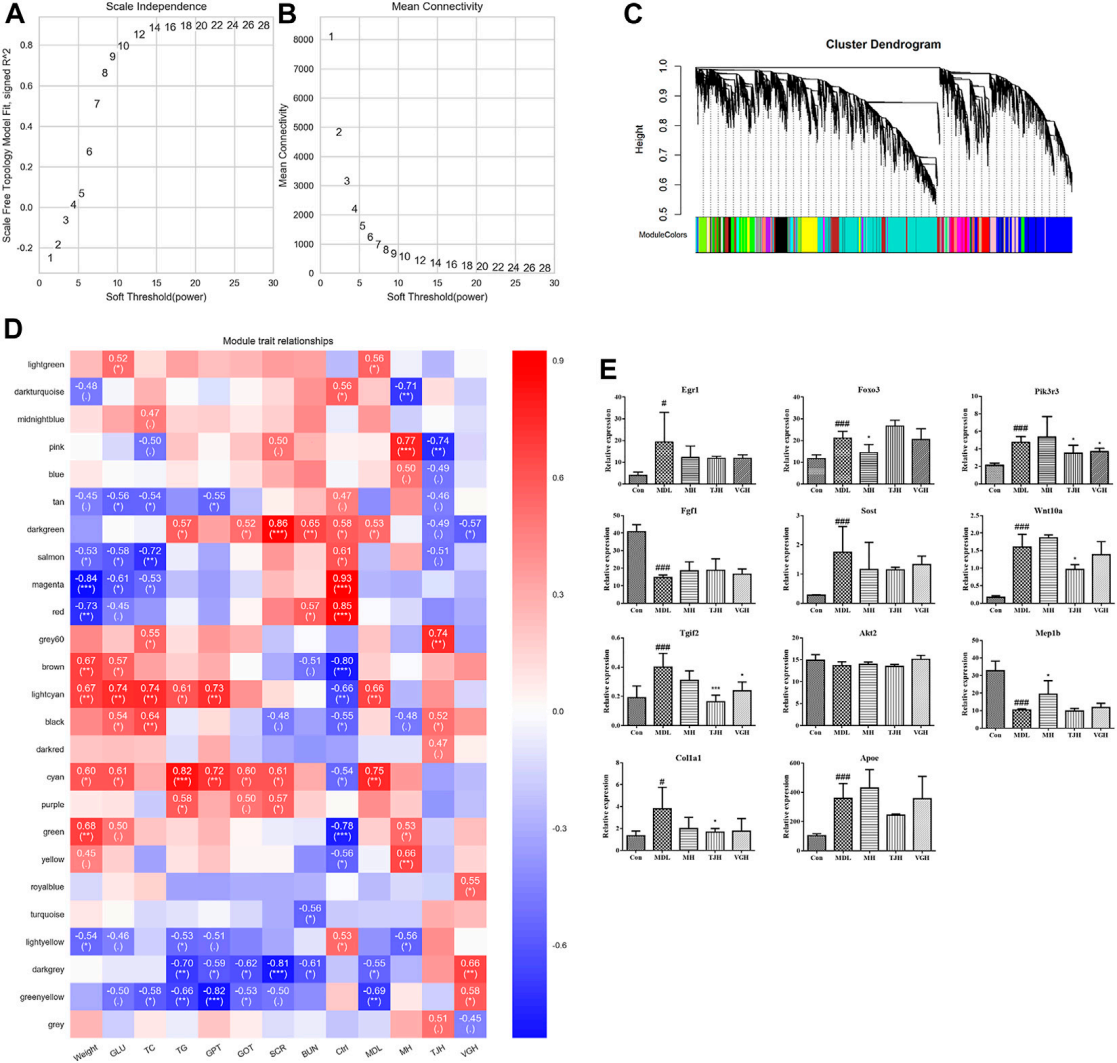
(**A**,**B)**. Power value filtering based on network; **(C)**. Genetic module classification; **(D)**. Heat map of correlation between module and biochemical index (”***”: *p* < 0.001; “**”: 0.001 ≤ *p* < 0.01; “*”: 0.01 ≤ *p* < 0.05; “.”: 0.05 ≤ *p* ≤ 0.1; *P*＞0.1, it will not be displayed); **(E)**. Relative expression of candidate genes different groups of samples (^#^ *p* < 0.05; ^##^ *p* < 0.01; ^###^ *p* < 0.001: models vs control; ^*^ *p* < 0.05; ^**^ *p* < 0.01; ^***^ *p* < 0.001: treatment groups vs. models). Real-time fluorescence quantitative PCR validation of candidate genes.

As an efficient and accurate bioinformatics and biological data mining method, weighted gene co-expression network algorithm (WGCNA) has been continuously improved and applied broadly (Zhang and Horvath, 2005). Here, the differential genes found at the transcriptome were correlated with the biochemical indicators that detected in the type 2 DKD model, and the gene network was further constructed. Other details see Supplementary Materials. The screening results of the power value of the network construction are shown in Supplementary Table S7and Figures 5A,B. Figure 5A showed the correlation coefficients corresponding to different powers, and Figure 5B showed the average connectivity of the network constructed with different power values, which showed that when the power was 14, the correlation coefficient was high, as well as the average connection degree of the network, so the power value used in the subsequent module construction was 14.

Based on the selected power value 14, a weighted co-expression network model was established and finally a total of 15,386 genes were divided into 25 modules by different colors. The gray module was a gene set that could not be attributed to any module, so it has no reference significance. The number of genes in the genes in each module is shown in Figure 5C, and the upper part of the figure is the gene clustering tree constructed by the dissTOM matrix constructed by the weighted correlation coefficient. The lower part of the Figure 5C shows the distribution of each module gene and the same color represents the same module. If the module features of the two different modules are genetically similar, they are automatically merged.

The P*earson* correlation algorithm was used to calculate the correlation coefficient and *p* value of the module characteristic genes and biochemical indicators. In this experiment, the biochemical indicators were correlated with genes, including weight, fasting blood glucose level after 8 weeks of administration, levels of triglyceride, total cholesterol, serum creatinine and serum urea nitrogen. The heat map can be seen in Figure 5D. In Figure 5D, the vertical axis represents each module and the horizontal axis represents each trait. The result shows the correlation between the module and the trait.

We found that the two modules with significant and inconsistent correlation trends were lightcyan module and cyan module; there are nine modules with significant correlation with the blank group, including tan, salmon, magenta, red, brown, black, green, yellow and lightyellow module. There were three modules that were significantly related to the db/db model group, including lightgreen, darkgrey and greenyellow module. Based on the significance of different modules, we queried the top 50 genes in each screening module for their association with DKD in combination with the network gene database (https://www.ncbi.nlm.nih.gov/gene/). A total of 11 candidate genes were identified, including Egr1, Foxo3, Pik3r3, Fgf1, Sost, Wnt10a, Tgif2, Akt2, Mep1b, Col1a1, and Apoe (Figure 5E), which were mainly key genes in TGF-β/Smad and PI3K/Akt/FoxO signaling pathway. The detailed parameters of the genes can be seen in Table 1.

**TABLE 1:** Candidate genes.

gene_id	baseMean	baseMean_control_Control	baseMean_case_Model	Fold change	log2FoldChange	P val	P adj	Up/Down	Control	Model
Egr1	629.8171	231.0385	1028.596	4.452053	2.154471	0.040332	0.240131	Up	4.06 ± 1.43	19.32 ± 13.64
Foxo3	2129.619	1566.554	2692.684	1.718859	0.781451	4.47E^−07^	2.89E^−05^	Up	11.74 ± 1.62	21.17 ± 3.1
Pik3r3	422.1827	270.857	573.5083	2.117384	1.082283	1.33E^−09^	1.62E^−07^	Up	2.14 ± 0.24	4.73 ± 0.67
Fgf1	3978.509	5919.504	2037.514	0.344203	-1.53867	6.89E^−25^	5.84E^−22^	Down	40.84 ± 3.96	14.7 ± 1.45
Sost	42.59663	13.75403	71.43923	5.19406	2.376863	0.000292	0.006904	Up	0.28 ± 0.01	1.75 ± 0.88
Wnt10a	25.99778	5.397115	46.59845	8.633956	3.110022	1.55E^−09^	1.84E^−07^	Up	0.18 ± 0.04	1.6 ± 0.36
Tgif2	34.24088	22.41959	46.06217	2.05455	1.038822	0.009295	0.090277	Up	0.19 ± 0.08	0.4 ± 0.09
Akt2	1636.926	1752.81	1521.042	0.867773	-0.20461	0.18374	0.566908	Down	14.89 ± 1.27	13.66 ± 0.89
Mep1b	1186.108073	1815.089162	557.126985	0.306941938	-1.703962318	3.32E-14	1.00E^−11^	Down	32.73 ± 5.51	10.42 ± 0.59
Col1a1	283.145249	157.3987414	408.8917565	2.597808297	1.377294972	0.031493193	0.205445201	Up	1.34 ± 0.43	3.8 ± 1.95
Apoe	7173.173801	3315.058638	11031.28897	3.327630118	1.73449508	2.12E-05	0.000800668	Up	103.07 ± 13.9	359.13 ± 101.38

### Real-time fluorescence quantitative PCR validation of candidate genes

The results of WGCNA analysis were further verified by real-time PCR quantification. The experimental results were based on the expression of the blank group and the difference between the disease group and the blank group was evaluated by FC-value and *p*-value. FC > 1.5 or FC < 0.67 indicated a significant difference between experimental group and blank group. The verification results are listed in Table 2. Except for Akt2 gene, the FC-value of other candidate genes had a significant difference between disease group and blank group; the *p*-value of Foxo3, Pik3r3, Fgf1, Wnt10a, and Mep1b genes showed a significant difference.

**TABLE 2:** Candidate genes validation.

Gene	FC-Value con vs. MDL	P-Value con vs. MDL
Egr1	3.822322	0.175687
Foxo3	1.599497	0.00031
Pik3r3	1.551625	0.002654
Fgf1	0.295552	0.018858
Sost	2.850806	0.065136
Wnt10a	4.653438	0.014783
Tgif2	1.6666	0.060593
Akt2	0.75556	0.084682
Mep1b	0.293199	0.010849
Col1a1	2.293331	0.159263
Apoe	2.54738	0.105881

After the treatment of multi-bioactive compounds combination of *Salvia miltiorrhiza*, the differential candidate genes were changed to varying degrees (Table 3). Metformin and multi-bioactive compounds combination from *Salvia miltiorrhiza* may regulate different molecular pathways to treat DKD. The positive drug group could significantly change the Foxo3, Fgf1, Sost, Akt2, and Mep1b genes. TJ group and VG group could significantly regulate the Egr1, Pik3r3 and Col1a1 genes.

**TABLE 3:** Regulation of multi-bioactive compounds combination on differential candidate genes.

Gene	FC-Value			P-Value		
	MDL vs. MH	MDL vs. TJH	MDL vs. VGH	MDL vs. MH	MDL vs. TJH	MDL vs. VGH
Egr1	0.705968	0.549043	0.500241	0.518296	0.341142	0.303367
Foxo3	0.851511	1.1245	0.877599	0.002549	0.072078	0.316091
Pik3r3	1.299359	0.744304	0.82166	0.075605	0.005148	0.020719
Fgf1	1.482029	1.283115	1.061877	0.011011	0.251666	0.665722
Sost	0.627235	0.782331	0.871475	0.272875	0.340254	0.554061
Wnt10a	1.577122	0.736772	0.95392	0.148055	0.224915	0.848578
Tgif2	1.033512	0.884769	0.956578	0.821024	0.614892	0.803241
Akt2	1.143723	1.007503	1.04592	0.014682	0.784118	0.471872
Mep1b	2.069585	0.929148	0.927403	0.104949	0.471726	0.479246
Col1a1	0.870926	0.478604	0.642959	0.686801	0.184324	0.416325
Apoe	1.287144	0.84279	1.163685	0.318679	0.545231	0.678083

### Proteomics alternations

According to the score of Score Sequest HT > 0 and unique peptide ≥1 with the blank value removed, the screening results were as the following: 3,104 reliable proteins were found in the trusted protein 1; 3,107 trusted proteins were identified in letter protein 2; 3,103 trusted proteins were discovered in authentic protein 3. There were 2,637 trusted proteins identified in the three groups.

Based on the selected trusted proteins, the results are combined using the index function for differential screening. *T*-test was performed for three replicate values of each group to calculate the difference fold FC value and the difference significance *p*-value of each comparison group. Then the differentially significant protein was screened by FC > 1.2 or FC < 5/6 and *p*-value < 0.05. After comparing the blank group with the disease group, 539 significant differential proteins were identified (Supplementary Figure S3).

Based on the two related pathways predicted and verified by combining transcriptome and WGCNA analysis results of differentially expressed genes, 11 candidate differential proteins were selected, including A8DUK4 (Beta-globin), A2ARV4 (Low-density lipoprotein receptor-related protein 2), Q91Y97 (Fructose-bisphosphate aldolase B), Q91VB8 (Alpha-globin), Q9DBM2 (Peroxisomal bifunctional enzyme), P09411 (Phosphoglycerate kinase 1), P26443 (Glutamate dehydrogenase 1, mitochondrial), S4R1W1 (Glyceraldehyde-3-phosphate dehydrogenase), P47738 (Aldehyde dehydrogenase, mitochondrial), A0A087WS56 (Fibronectin), and P52503 (NADH dehydrogenase [ubiquinone] iron-sulfur protein 6, mitochondrial), which correspond to the genes are Hbb-bs, Lrp2, Aldob, Hba-a1, Ehhadh, Pgk1, Glud1, Gm3839, Aldh2, Fn1, and Ndufs6, respectively.

### PRM targeted quantitative proteome analysis validation of candidate proteins

**FIGURE 6:**
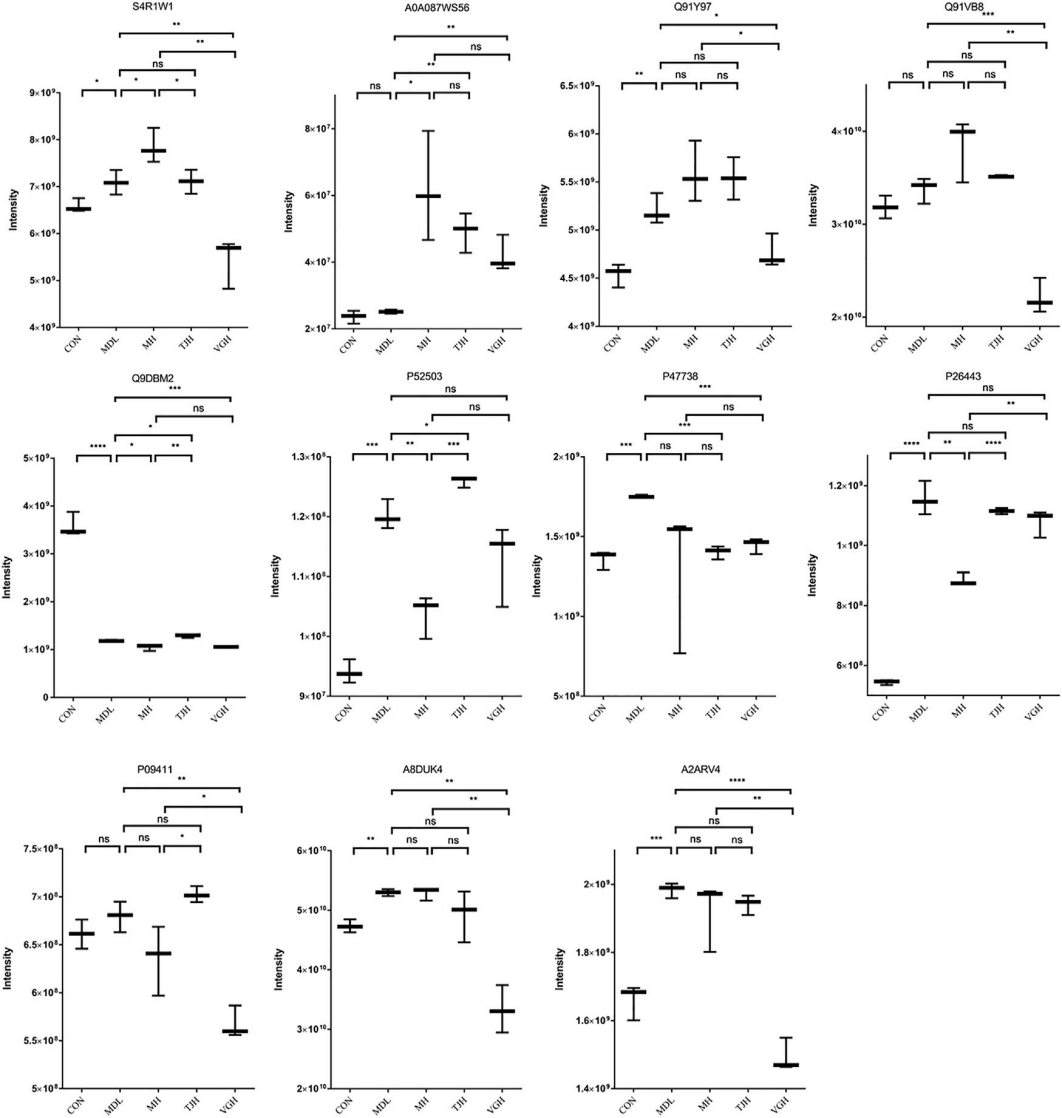
Quantitative comparison of target proteins. (ns: *P* ＞ 0.05, not significant; ^#^ *p* < 0.05; ^##^ *p* < 0.01; ^###^ *p* < 0.001: models vs. control; *:*p* < 0.05; **: *p* < 0.01; ***: *p* < 0.001; ****: *p* < 0.0001)

Parallel Reaction Monitoring (PRM) is a derivative technology of Selected Reaction Monitoring (SRM). The entire fragment ion map of each target parent ion can be continuously recorded throughout the liquid phase separation process by PRM technology. Compared to SRM which only detects the pattern of the target ion pair, PRM detects all fragment information in the selected parent ion window (Peterson et al., 2012; Ronsein et al., 2016). The quantitative information of the target peptide is derived, and the quantitative value of the protein is calculated by peptide addition and used for statistical analysis between groups (Cox and Mann, 2008). The fold change was calculated using the ratio of experimental and control groups and the genes corresponding to each protein are listed in the Table 4. As shown in Figure 6, except for Q91VB8, P09411, and A0A087WS56 proteins, the other proteins in the disease group have shown a significant difference, compared with the control group. The positive drug metformin could significantly callback P52503 and P26443 protein; TJH could significantly callback Q9DBM2 and P47738 protein; VGH could significantly callback S4R1W1, Q91Y97, P47738, A8DUK4 and A2ARV4 protein, which showed that different drugs could improve the progress of the disease by regulating different proteins.

**TABLE 4:** Relative quantitative results of target proteins.

Protein ID	Protein	Gene	Fold change					
			MDL/CON	MH/MDL	TJH/MDL	VGH/MDL	MH/TJH	MH/VGH
A0A087WS56	Fibronectin	Fn1	1.10	1.49	1.17	0.64	1.27	2.31
A2ARV4	Low-density lipoprotein receptor-related protein 2	Lrp2	1.21	0.96	0.94	0.74	1.02	1.30
A8DUK4	Beta-globin	Hbb-bs	1.12	1.00	0.92	0.62	1.09	1.62
P09411	Phosphoglycerate kinase 1	Pgk1	1.03	0.94	1.03	0.83	0.90	1.12
P26443	Glutamate dehydrogenase 1, mitochondrial	Glud1	2.12	0.77	0.97	0.93	0.79	0.82
P47738	Aldehyde dehydrogenase, mitochondrial	Aldh2	1.31	0.88	0.83	0.82	1.06	1.08
P52503	NADH dehydrogenase [ubiquinone] iron-sulfur protein 6, mitochondrial	Ndufs6	1.28	0.86	1.05	0.94	0.82	0.92
Q91VB8	Alpha globin 1	Hba-a1	1.07	1.15	1.12	0.72	1.02	1.59
Q91Y97	Fructose-bisphosphate aldolase B	Aldob	1.14	1.06	1.06	0.96	1.00	1.11
Q9DBM2	Peroxisomal bifunctional enzyme	Ehhadh	0.33	0.89	1.09	0.88	0.81	1.02
S4R1W1	Glyceraldehyde-3-phosphate dehydrogenase	Gm3839	1.08	1.11	1.00	0.77	1.10	1.45

## Discussion

Arachidonic acid metabolism is mainly involved in chronic inflammatory responses (Soto et al., 2018). Based on the results of biochemical indicators and metabolomics, we speculated that long-term high blood glucose levels *in vivo* would affect the expression of oxidative kinases and protein kinases *in vivo*, thereby modulating the expression of growth and transformation factors, leading to abnormal levels of biochemical indicators, and at the same time causing chronic inflammation and metabolic disorders, such as arachidonic acid and fatty acid metabolic disorders. Based on the results of transcriptomics, the pathogenesis of diabetic kidney disease may involve 11 candidate differential genes: Egr1, Foxo3, Pik3r3, Fgf1, Sost, Wnt10a, Tgif2, Akt2, Mep1b, Col1a1, Apoe. Among them, Foxo3, Pik3r3, Fgf1, Akt2, and Col1a1 are associated with PI3K/AKT/FoxO signaling pathway, and Tgif2 is related to TGF-β/Smad signaling pathway (https://www.kegg.jp). According to the results of transcriptomic, 11 related differential proteins involved in the TGF-β/Smad and PI3K/Akt/FoxO signaling axis were selected and verified by proteomics, including A8DUK4 (Beta-globin), A2ARV4 (Low-density lipoprotein receptor-related protein 2), Q91Y97 (Fructose-bisphosphate aldolase B), Q91Y97, Q91VB8 (Alpha-globin), Q9DBM2 (Peroxisomal bifunctional enzyme), P09411 (Phosphoglycerate kinase 1), P26443 (Glutamate dehydrogenase 1, mitochondrial), S4R1W1 (Glyceraldehyde-3-phosphate dehydrogenase), P47738 (Aldehyde dehydrogenase, mitochondrial), A0A087WS56 (Fibronectin), and P52503 (NADH dehydrogenase [ubiquinone] iron-sulfur protein 6, mitochondrial).

According to the experimental results, the therapeutic effect of TJ and VG group was generally similar to that of positive drug, which preliminarily verified that multi-bioactive compounds combination from *Salvia miltiorrhiza* had a good therapeutic effect on DKD. After treatment by multi-bioactive compounds combination from *Salvia miltiorrhiza*, the differential candidate genes and proteins have changed. Based on the results of multi-omics and verification experiments, the molecular mechanism of multi-bioactive compounds combination from *Salvia miltiorrhiza* for improving DKD was predicted, which involved the TGF-β/Smad and PI3K/Akt/FoxO signaling axis.

Col1a1 (collagen type I α1 chain) is a protein-coding gene whose associated pathways include angiotensin activation of ERK and collagen-chain trimerization (Ortuño et al., 2013). The GO annotation associated with this gene includes the same protein binding and platelet-derived growth factor binding. In the previous experiments, we can also find that the expression levels of E-cadherin and TGF-β1 in the model group were significantly changed. TGF-β1 stimulates the expression of Col1a1 and phosphorylation of phosphoinositide three kinase (PI3k) and Akt (Foretz et al., 2010; Yang et al., 2013). E-cadherin can induce the activation of PI3k/Akt signaling in cells, and down-regulates early growth response gene 1 (Egr1) by inhibiting phosphatase and angiotensin homologues (Lau et al., 2011).

High glucose level in the body also affects the expression of Q91Y97 protein (gene Aldob), which can further affect glycolysis gluconeogenesis, pentose phosphate pathway and tricarboxylic acid (TCA) cycle (Zhou et al., 2020; Potter et al., 2021). Q91Y97 protein is involved in step of the subpathway that synthesizes D-glyceraldehyde 3-phosphate and glycerone phosphate from D-glucose. The knockdown of Q91Y97 protein (gene Aldob) expression can prevent fructose-induced methylglyoxal overproduction and vascular smooth muscle cell proliferation. Moreover, fructose significantly increased carbohydrate-responsive element-binding protein (ChREBP), phosphorylated FoxO1/3α and Akt1 levels (Cao et al., 2017). P26443 protein (gene Glud1) is mitochondrial glutamate dehydrogenase that converts L-glutamate into alpha-ketoglutarate, which plays a key role in glutamine anaplerosis by producing alpha-ketoglutarate and is an important intermediate in the TCA cycle (Csibi et al., 2013). Also, it may be involved in learning and memory reactions by increasing the turnover of the excitatory neurotransmitter glutamate and can contribute to glucose-stimulated insulin secretion in murine β-cells, but not to basic insulin release (Petraki et al., 2019; Roy et al., 2019).

Lrp2 is a type of macromembrane glycoprotein and belongs to the family of low-density lipoprotein receptor proteins. Megalin, an endocytic receptor, is thought to be an important component of many pathological conditions, including diabetic nephropathy. The expression of megalin may be severely compromised in disease states, and the mechanism may be related to activation of the renin-angiotensin system, increased TGF-β signaling, etc (Marzolo and Farfán, 2011). The results showed that Lrp2 was significantly increased in db/db mice, and the VG group could be significantly recalled.

**FIGURE 7:**
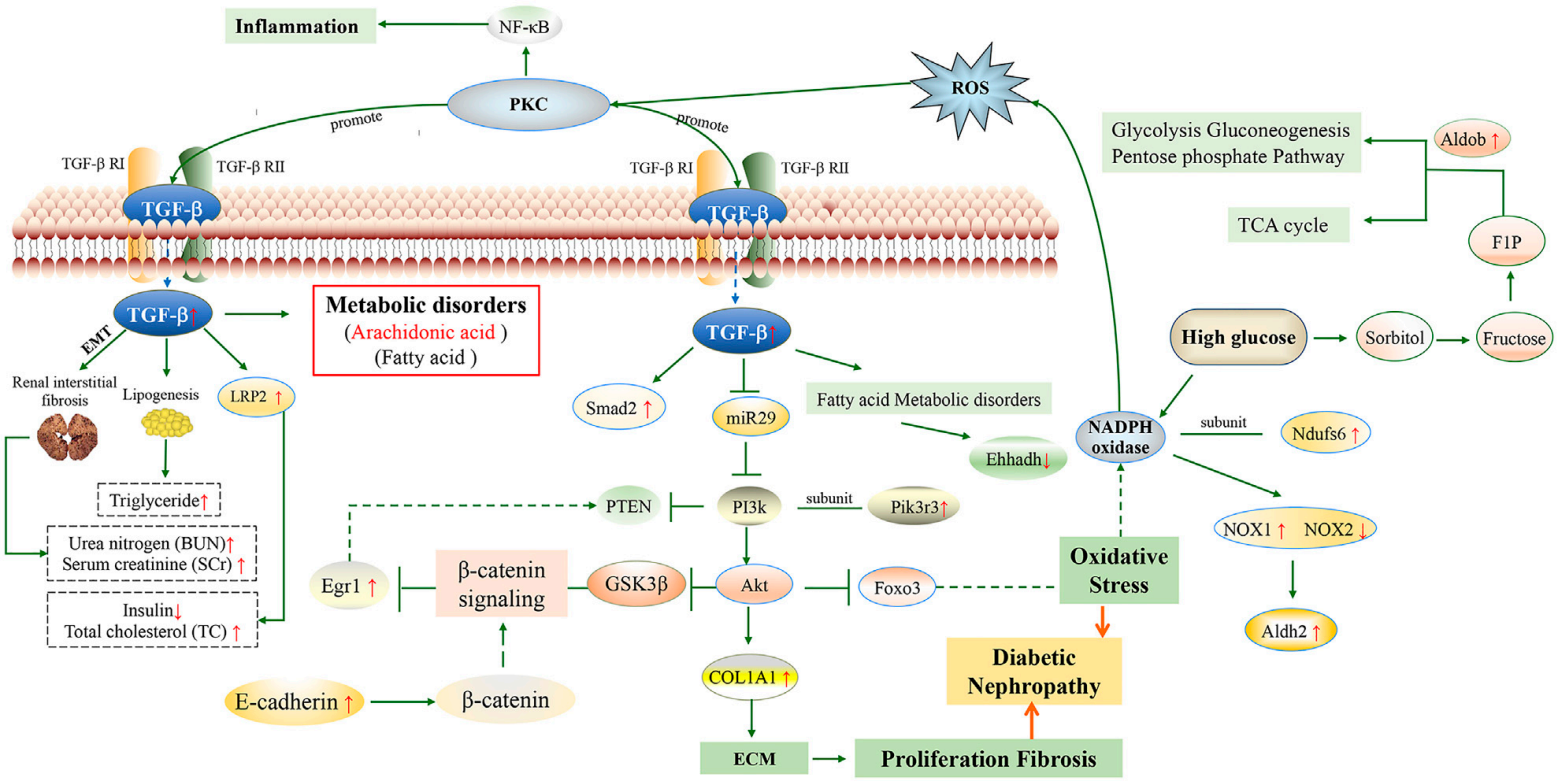
Molecular mechanism of multi-bioactive compounds combination from *Salvia miltiorrhiza* to improve DKD. (The red arrows represent changes in db/db model mice).

From the factors mentioned above, we may summarize the molecular mechanism of multi-bioactive compounds combination from *Salvia miltiorrhiza* for improving DKD. Multi-bioactive compounds combination may improve DKD by regulating TGF-β/Smad and PI3K/Akt/FoxO signaling pathways and abnormal protein expression, thereby affecting the process of oxidative stress, ECM collagen deposition and kidney tissue fibrosis (Figure 7). Pathological deposition of collagen is identified as a hallmark of kidney fibrosis (Baues et al., 2020). High glucose level in the body also affects the expression of Q91Y97 protein, which can further affect glycolysis gluconeogenesis, pentose phosphate pathway and TCA cycle.

In this experiment, we first time revealed the pathogenesis of DKD via multi-omics approaches. The pathogenesis of DKD may involve 11 candidate differential genes and TGF-β/Smad and PI3K/Akt/FoxO signaling pathways. The multi-bioactive compounds combination of *Salvia miltiorrhiza* may ameliorate kidney injury in diabetes through downregulation of the TGF-β/Smad and PI3K/Akt/FoxO signaling pathways and ameliorating oxidative stress, ECM collagen deposition, and kidney tissue fibrosis. Our study may therefore provide a scientific basis and support for the clinical diagnosis of DKD and therapeutic explorations to tackle this devastating disorder.

## Data Availability

Mass spectrometry proteomics data has been deposited into the ProteomeXchange Consortium (http://proteomecentral.proteomexchange.org/cgi/GetDataset?ID=PXD036837) with the dataset identifier PXD036837. Other data that support the findings of this study are available from the corresponding author upon reasonable request.
