# Supplementary material for: Multi-omics analysis reveals the pathogenesis of db/db mice diabetic kidney disease and the treatment mechanisms of multi-bioactive compounds combination from Salvia miltiorrhiza
Source: Front Pharmacol. 2022 Sep 29;13:987668. doi: 10.3389/fphar.2022.987668 (PMC9557128; doi:10.3389/fphar.2022.987668)
Supplement: Supplementary file 1 [file DataSheet1.docx]

Supplementary Material

# Metabolomics study on serum and urine samples (Dai et al., 2018)

Chromatographic separation was performed by using a Waters Acquity^TM^ Ultra Performance LC system (Waters Corp.) equipped with a Waters Xevo^TM^ G2 Q/TOF-MS (Waters Corp.). Acquity UPLC BEH C_18_ (100 mm × 2.1 mm, 1.7 *μ*m, Waters Corp.) was applied for all analyses at 35 °C and the flow rate was 0.4 mL·min^-1^. Chromatographic analysis was performed with gradient elution using water containing 0.1% formic acid (A) and acetonitrile (B). Serum samples analysis conditions were as the following: 0~3 min, 95%~55% A; 4~13 min, 55%~5% A; 13~14 min, 5% A. For urine samples analysis, the optimized UPLC elution conditions were as the following: 0~8 min, 95%~70% A; 8~11 min, 70%~30% A; 11~13 min, 30%~5% A; 13~14 min, 5% A. The autosampler temperature was 4 °C.

Mass spectrometry analysis was performed on a Waters SynaptTM QTOF/MS spectrometer (Waters Corp.) with an electrospray ionization source in positive and negative ionization modes. The detailed parameters were as follows: extraction cone voltage 2.0 V, sample cone voltage 30.0 V, capillary voltage 3.0 kV, desolvation temperature 400 °C, source temperature 120 °C. Nitrogen was used as the cone and desolvation gas at flow rates of 50 and 600 L/h, respectively. Metabolomics in centroid mode was set from 100 to 1,000 Da. Leucine-enkephalin was chosen as the locked mass solution to generate [M+H]^+^ ion (*m/z* 556.2771) and [M-H]^-^ ion (*m/z* 554.2615) in positive and negative modes to ensure accuracy,. The data acquisition rate was set to 30 ms and the inter-scan delay was 0.02 s.

10 randomly selected serum samples were mixed as the quality control (QC) samples to optimize the condition of UPLC-QTOF/MS. The QC samples was injected six times at the beginning of running to condition or equilibrate the system and then every 8 samples to further monitor the stability of analysis. After the instrument was calibrated, the QC sample was firstly analyzed daily to test the stability of the instrument to ensure consistent performance of the system.

# Proteomics study on kidney tissues

Mobile phases A (2% acetonitrile in HPLC water) and B (98% acetonitrile in HPLC water) were used for reversed-phase gradient analysis of protein separation. The solvent gradient was set as the following: 0~8 min, 98% A; 8.00~8.01 min, 98%~95%A; 8.01~38 min, 95%~75% A; 38~50 min, 75~60% A; 50~50.01 min, 60~10% A; 50.01~60 min, 10% A; 60~60.01 min, 10~98% A; 60.01~65 min, 98%A.

The lyophilized peptide fractions were re-suspended in 2% acetonitrile containing 0.1% formic acid, and loaded into a C_18_ (75 *μ*m × 150 mm, 2 *μ*m) trap column with flow rate of 300 nL/min. Mobile phases A (0.1% formic acid in HPLC water) and B (80% acetonitrile and 0.1% formic acid in HPLC water) were used for reversed-phase gradient. The solvent gradient was set as the following: 0~55 min, 8% B; 55~79min, 30% B; 79~80 min, 50% B; 80~90 min, 100% B. The primary MS mass resolution was set to mass resolution set to 70,000 and the automatic gain control value is set to 1e^6^. The mass spectrometry scan was set to the full scan charge-to-mass ratio *m/z* range 300-1600 and MS/MS scans were performed on the highest ten peaks. All MS/MS spectra were acquired using high-energy collisional cleavage in positive ion mode and spectral acquisition using high-energy collisional cleavage in a data-dependent positive ion mode completion. The collision energy is set to 32, the resolution of MS/MS is set to 17,500, the automatic gain control is set to 2e^5^, the maximum accumulation time of ions is 80 ms, and the exclusion time is set to 15 s.

# PRM targeted quantitative proteome analysis

A 90 minutes' gradient was performed as the following: 0~60 min, 8~24%B; 60~79 min, 24~45%B; 79~80 min, 45~100% B; 80~90 min, 100% B. Peptides were then transferred to the gaseous phase with positive ion electrospray ionization at 2.1kV. For DDA, the top10 precursors were acquired between 350 and 1650 *m/z*, dynamic exclusion of 40 seconds, normalized collision energy of 27. Resolution for MS1 was 120,000 and MS2 was 30,000. For PRM, precursors were targeted in a 1.2 *m/z* isolation window around the *m/z* of interest. Precursors were fragmented in HCD mode with NCE energy of 32. MS/MS was performed at 30,000 resolutions and AGC target of 5e^5^and a maximum injection time was 200 ms. Spectra were analyzed using Skyline with manual validation[30]. Finally, the original files were imported into the Proteome Discover 2.3 software for searching. The main parameters are set as the following, fixed modification: Carbamidomethyl (C); variable modification: Oxidation (M); MS1: 10 ppm; MS2: 0.02Da; missed cleavage: 2; enzyme: trypsin; database: uniprot-proteome_ UP000000589-Mus musculus (Mouse) (Strain C57BL6J). *fasta*.

# Weighted gene co-expression network analysis

In order to satisfy the precondition of scale-free network distribution as far as possible, it is necessary to select the value of adjacency matrix weight parameter power in data analysis. The power value was set from 1 to 30, and the correlation coefficient and the average connectivity of the network were calculated, respectively. The higher the correlation coefficient (maximum 1), the closer the network was to scale-free network distribution, but we also assured a certain degree of gene connectivity, so the power value was large enough to ensure the gene connectivity.

**Table S1** Subgrouping and administration (n = 10)

| No. | Group | Dosage (mg/kg·d) | No. | Group | Dosage (mg/kg·d) |
| --- | --- | --- | --- | --- | --- |
| 1 | Control (Con) | Solvent | 9 | Low dose of FJ (FJL) | 100 |
| 2 | Model (MDL) | Solvent | 10 | High dose of FG (FGH) | 200 |
| 3 | Metformin (MH) | 250 | 11 | Low dose of FG (FGL) | 100 |
| 4 | High dose of TJ (TJH) | 200 | 12 | High dose of VJ (VJH) | 200 |
| 5 | Low dose of TJ (TJL) | 100 | 13 | Low dose of VJ (VJL) | 100 |
| 6 | High dose of TG (TGH) | 200 | 14 | High dose of VG (VGH) | 200 |
| 7 | Low dose of TG (TGL) | 100 | 15 | Low dose of VG (VGL) | 100 |
| 8 | High dose of FJ (FJH) | 200 |  |  |  |

**Table S2** Multi-bioactive compounds combination compatibility proportion (mass ratio)

| Group | Salvianolic acid B | Rosmarinic acid | Lithospermic acid | Danshensu | Tanshinone ⅡA |
| --- | --- | --- | --- | --- | --- |
| Three monomers combined with ratio of stem-leaf (TJ) | 1 | 0.58 | 0.01 | - | - |
| Three monomers combined with ratio of root (TG) | 1 | 0.03 | 0.03 | - | - |
| Four monomers combined with ratio of stem-leaf (FJ) | 1 | 0.58 | 0.01 | 0.03 | - |
| Four monomers combined with ratio of root (FG) | 1 | 0.03 | 0.03 | 0.02 | - |
| Five monomers combined with ratio of stem-leaf (VJ) | 1 | 0.58 | 0.01 | 0.03 | 0.02 |
| Five monomers combined with ratio of stem-leaf (VG) | 1 | 0.03 | 0.03 | 0.02 | 0.02 |

The “-” represents not add.

**Table S3** Volume and concentration of libraries and quality of raw sequence reads

| Sample | Library Concentration(ng/μL) | Volume(μL) | Q30 |
| --- | --- | --- | --- |
| Sample_Con1 | 20.4 | 15 | 94.52% |
| Sample_Con2 | 25.6 | 15 | 94.80% |
| Sample_Con3 | 21.4 | 15 | 93.78% |
| Sample_MDL1 | 26.6 | 15 | 94.51% |
| Sample_MDL2 | 24.2 | 15 | 94.70% |
| Sample_MDL3 | 24.8 | 15 | 94.50% |
| Sample_MH1 | 26.6 | 15 | 94.51% |
| Sample_MH2 | 14.5 | 15 | 94.34% |
| Sample_MH3 | 26.8 | 15 | 94.59% |
| Sample_TJH1 | 28 | 15 | 94.68% |
| Sample_TJH2 | 28.4 | 15 | 94.19% |
| Sample_TJH3 | 31 | 15 | 94.21% |
| Sample_VGH1 | 24.8 | 15 | 94.37% |
| Sample_VGH2 | 28.4 | 15 | 94.57% |
| Sample_VGH3 | 27.6 | 15 | 94.43% |

**Table S4** The primer sequences

| **Gene Symbol** | **Forward primer (5' to 3')** | **Reverse primer (5' to 3')** | **Product length(bp)** |
| --- | --- | --- | --- |
| Egr1 | TTGCCTCCCATCACCTATAC | GGATTGGTCATGCTCACG | 119 |
| Foxo3 | GTGGACAGTGATCCGTTTAC | CAGTCTCTGCTGGGTTAGG | 72 |
| Pik3r3 | GGTGGAGCTTATTAACCACTAC | GATCCTGTTGGAATCTGGATAC | 101 |
| Fgf1 | AAACCACTGCACAAGGAT | GTTGACGGTTAAGGCTACAAT | 85 |
| Sost | GTGATGTTGGGCTACGTG | CAATCCTTGGAATCTCAGCAG | 84 |
| Wnt10a | CCCAGGAGTGATAGGCAA | GTCTGGAGCCCTTAGAGTC | 105 |
| Tgif2 | GCAGATGTCACAGAACCG | TGGGCTACCTACTGCATTAAC | 100 |
| Akt2 | TGGACCACAGTCATCGAG | GTTGGCGACCATCTGGATA | 87 |
| Mep1b | GGAGAAGACTGGTGGTACA | CGTGGAAGAAACAGCGAT | 84 |
| Col1a1 | CTGTGCCTCAGAAGAACT | GGAATCCATCGGTCATGC | 87 |
| Apoe | GTCCTGCAACAACATCCATA | GAATGTGCTCGGAGAATCTT | 100 |
| Gapdh | GCAAGGACACTGAGCAAGA | GGATGGAAATTGTGAGGGAG | 76 |

**Table S5** Potential biomarkers selected and identified between db/db mice and normal group in serum and urine

| Metabolites | m/z | Retention time (min) | Ion mode | Compound ID | Formula | VIP | *P*-value | adj. *P*-value | log2(FC) | Control | Model | Source |
| --- | --- | --- | --- | --- | --- | --- | --- | --- | --- | --- | --- | --- |
| PG(16:0/0:0) | 502.3134 | 7.106217 | pos | LMGP04050008 | C_22_H_45_O_9_P | 2.56159 | 0.000109 | 0.002663 | 0.973818 | 24667.22±6363.02 | 12559.49±1025.31 | serum |
| Leukotriene D4-d5 | 500.2848 | 7.0837 | neg | 96385 | C_25_H_35_D_5_N_2_O_6_S | 2.55516 | 0.000484 | 0.008092 | 1.445572 | 20639.51±8054.08 | 7577.73±1429.75 | serum |
| PC(18:1(11Z)/22:6(4Z,7Z,10Z,13Z,16Z,19Z)) | 832.5889 | 12.56332 | pos | HMDB0008090 | C_48_H_82_NO_8_P | 2.30552 | 0.030675 | 0.141629 | 1.870336 | 20648.58±16434.86 | 5647.58±6448.53 | serum |
| Glucosylsphingosine | 506.3342 | 8.242533 | neg | HMDB0000596 | C_24_H_47_NO_7_ | 2.29627 | 0.004481 | 0.038948 | -0.5281 | 29360.8±8709.97 | 42339.17±6483.28 | serum |
| Ecabet | 379.1637 | 11.31797 | neg | HMDB0015613 | C_20_H_28_O_5_S | 2.10711 | 0.048438 | 0.196409 | 0.991732 | 28346.32±18125.38 | 14254.61±3375.15 | serum |
| 12-Hydroxy-12-octadecanoylcarnitine | 426.3588 | 8.265317 | pos | HMDB0013154 | C_25_H_49_NO_5_ | 1.69713 | 7.12E-05 | 0.00206 | -0.88818 | 6891.34±1913.44 | 12754.8±2293.3 | serum |
| Decanoylcholine | 303.2402 | 11.08588 | neg | HMDB0013228 | C_15_H_32_NO_2_+ | 1.6002 | 0.018093 | 0.101081 | 0.517472 | 24527.3±6183.42 | 17134.65±4777.88 | serum |
| Phthalic acid | 149.0232 | 8.32905 | pos | HMDB0002107 | C_8_H_6_O_4_ | 1.38026 | 0.010838 | 0.070405 | -0.44923 | 14835.72±4105.93 | 20255.43±3224.58 | serum |
| PC(19:3(10Z,13Z,16Z)/0:0) | 530.3251 | 7.7375 | neg | LMGP01050003 | C_27_H_50_NO_7_P | 1.35999 | 0.000108 | 0.002663 | 1.642234 | 5533.45±1902.42 | 1772.7±614.12 | serum |
| LysoPC(22:5(4Z,7Z,10Z,13Z,16Z)) | 570.3581 | 7.506833 | pos | HMDB0010402 | C_30_H_52_NO_7_P | 1.34254 | 7.12E-05 | 0.00206 | 1.849101 | 4806.2±1703.6 | 1334.04±475.08 | serum |
| LysoPC(20:3(5Z,8Z,11Z)) | 590.3455 | 7.7375 | neg | HMDB0010393 | C_28_H_52_NO_7_P | 1.19998 | 0.000119 | 0.00271 | 1.805976 | 4093.87±1493.61 | 1170.8±483.63 | serum |
| 1-Arachidonoylglycerophosphoinositol | 619.2892 | 7.211133 | neg | HMDB0061690 | C_29_H_49_O_12_P | 1.17905 | 0.010354 | 0.068718 | 0.591162 | 9905.56±2633.64 | 6575.39±1787.46 | serum |
| LysoPE(0:0/20:4(5Z,8Z,11Z,14Z)) | 484.284 | 7.106217 | pos | HMDB0011487 | C_25_H_44_NO_7_P | 1.15455 | 1.98E-05 | 0.00086 | 1.513345 | 3805.53±1093.26 | 1333.07±199.87 | serum |
| PC(15:1(9Z)/0:0) | 478.2944 | 6.599817 | neg | LMGP01050125 | C_23_H_46_NO_7_P | 1.13806 | 0.000219 | 0.004328 | -0.86337 | 3083.47±1155.87 | 5609.7±871.87 | serum |
| Neotame | 377.2068 | 6.38935 | neg | HMDB0034566 | C_20_H_30_N_2_O_5_ | 10.2473 | 4.17E-08 | 2.72E-07 | -11.4486 | 34.97±54.91 | 97739.42±25906.01 | urine |
| 5S-HETE di-endoperoxide | 425.2245 | 12.8384 | pos | LMFA03000011 | C_20_H_34_O_8_ | 8.41201 | 1.16E-08 | 1.05E-07 | -0.70006 | 104244.33±12457.53 | 169352.56±9403.49 | urine |
| Rollinecin A | 663.4601 | 14.08092 | pos | HMDB0030438 | C_37_H_68_O_7_ | 5.8075 | 1.12E-07 | 5.98E-07 | -0.68915 | 50894.34±7140.54 | 82058.71±5389.44 | urine |
| Metenamine | 141.1138 | 13.32237 | pos | HMDB0029598 | C_6_H_12_N_4_ | 5.7283 | 7.92E-08 | 4.51E-07 | -0.63296 | 55432.6±7242.44 | 85961.65±4488.28 | urine |
| TG(17:2(9Z,12Z)/22:3(10Z,13Z,16Z)/22:6(4Z,7Z,10Z,13Z,16Z,19Z))[iso6] | 984.805 | 9.024283 | pos | LMGL03012146 | C_64_H_102_O_6_ | 5.18422 | 2.52E-09 | 3.33E-08 | 3.003096 | 28443.38±5020.78 | 3547.8±1706.33 | urine |
| Xanthurenic acid | 206.0454 | 2.175517 | pos | 5841 | C_10_H_7_NO_4_ | 4.98934 | 1.43E-07 | 7.38E-07 | 3.414043 | 26068.67±6897.75 | 2445.62±510.64 | urine |
| PIM1(17:0/16:1(9Z)) | 985.5865 | 9.024283 | pos | LMGP15010010 | C_48_H_89_O_18_P | 4.16536 | 1.56E-10 | 4.91E-09 | 2.039021 | 21105.93±2085.38 | 5135.68±1800.4 | urine |
| 1-O-(2R-hydroxy-heneicosanyl)-sn-glycerol | 822.7621 | 14.29182 | pos | LMGL01020067 | C_24_H_50_O_4_ | 4.07963 | 1.24E-06 | 4.55E-06 | -0.72989 | 24546.71±3396.83 | 40711.06±4534.51 | urine |
| beta-nicotinamide D-ribonucleotide | 380.0635 | 1.50165 | neg | HMDB0059645 | C_11_H_16_N_2_O_8_P+ | 4.0142 | 7.04E-09 | 7.16E-08 | 3.292782 | 16662.41±3399.52 | 1700.25±586.45 | urine |
| 5-Hydroxyindol-2-carboxylic acid | 178.0501 | 2.175517 | pos | 44729 | C_9_H_7_NO_3_ | 3.9107 | 5.76E-08 | 3.56E-07 | 4.085282 | 15303.38±3908.75 | 901.56±269.21 | urine |
| 6-hydroxy-2H-1,3-benzodioxole-5-carboxylic acid | 382.0782 | 1.52315 | pos | HMDB0129371 | C_8_H_6_O_5_ | 3.18563 | 1.13E-09 | 1.87E-08 | 7.646918 | 9390.46±1870.09 | 46.85±76.61 | urine |
| Isohumulone A | 363.2181 | 9.045533 | pos | HMDB0030026 | C_21_H_30_O_5_ | 3.17986 | 7.68E-06 | 2.26E-05 | -30.3586 | 0±0 | 10230.59±4211.78 | urine |
| Pelargonidin 3-(6''-p-coumarylsambubioside)-5-(6'''-malonylglucoside) | 982.2323 | 8.960467 | pos | LMPK12010055 | C_44_H_47_O_24_+ | 2.9722 | 1.19E-09 | 1.93E-08 | 5.171765 | 8330.39±1578.67 | 231.1±402.04 | urine |
| Thiazinamium | 344.1567 | 10.30733 | neg | HMDB0240235 | C_18_H_23_N_2_S+ | 2.93821 | 8.48E-08 | 4.75E-07 | 3.156634 | 9297.29±2229.52 | 1042.59±629.64 | urine |
| (S,E)-Zearalenone | 301.1432 | 12.39528 | pos | HMDB0031752 | C_18_H_22_O_5_ | 2.89651 | 4.65E-07 | 1.93E-06 | -0.69387 | 12822.41±1759.58 | 20741.69±1852.57 | urine |
| 12alpha-Hydroxy-13,18-dehydroparain | 375.183 | 5.86285 | neg | HMDB0039557 | C_21_H_28_O_6_ | 2.89385 | 2.55E-06 | 8.42E-06 | -8.65684 | 20.29±10.06 | 8188.57±3048.01 | urine |
| 5-Thymidylic acid | 321.0499 | 7.084083 | neg | HMDB0001227 | C_10_H_15_N_2_O_8_P | 2.72338 | 0.044294 | 0.060094 | 0.45032 | 46500.3±7666.81 | 34032.65±13997.04 | urine |
| 5-Hydroxy-2-furoic acid | 129.0196 | 12.8384 | pos | HMDB0059784 | C_5_H_4_O_4_ | 2.54682 | 1.28E-06 | 4.67E-06 | -0.72155 | 9472.48±1303.5 | 15619.7±1723.14 | urine |
| 1-Isothiocyanatohexane | 144.0846 | 2.239333 | pos | HMDB0038432 | C_7_H_13_NS | 2.52479 | 2.37E-10 | 6.54E-09 | 3.450348 | 6362.43±1004.09 | 582.06±224.15 | urine |
| 1-Fluorocyclohexadiene-cis,cis-1,2-diol | 131.0502 | 5.86395 | pos | 71225 | C_6_H_7_FO_2_ | 2.48694 | 1.82E-06 | 6.31E-06 | 0.914542 | 12707.44±1601.91 | 6741.45±1451.01 | urine |
| N(omega)-Hydroxyarginine | 235.104 | 14.66865 | neg | HMDB0004224 | C_6_H_14_N_4_O_3_ | 2.41107 | 1.03E-07 | 5.59E-07 | -0.88007 | 6368.61±1031.82 | 11721.23±1124.78 | urine |
| gamma-Glutamylphenylalanine | 275.1023 | 7.842367 | neg | HMDB0000594 | C_14_H_18_N_2_O_5_ | 2.40246 | 0.016169 | 0.023922 | 8.501354 | 8705.6±8984 | 24.02±15.47 | urine |
| 10Z-Pentacosene | 368.4263 | 13.53325 | pos | LMFA11000068 | C_25_H_50_ | 2.37861 | 7.62E-08 | 4.40E-07 | -0.66537 | 8965.9±1211.43 | 14219.67±818.16 | urine |
| TG(15:0/17:1(9Z)/15:0) | 796.7442 | 14.58777 | pos | LMGL03012614 | C_50_H_89_D_5_O_6_ | 2.33117 | 4.49E-06 | 1.39E-05 | -0.77221 | 7666.14±1794.37 | 13092.88±1144.98 | urine |
| Heptaethylene glycol | 325.1863 | 13.8481 | neg | HMDB0061835 | C_14_H_30_O_8_ | 2.23601 | 0.00338 | 0.00567 | -0.55033 | 12564.86±2674.56 | 18400.21±3847.09 | urine |
| Eicosanoyl-EA | 378.3328 | 13.40733 | pos | LMFA08040038 | C_22_H_45_NO_2_ | 2.13936 | 4.34E-07 | 1.82E-06 | -0.54933 | 9365.95±1321.4 | 13706.18±438.73 | urine |
| Histamine | 112.0874 | 13.32237 | pos | HMDB0000870 | C_5_H_9_N_3_ | 2.12516 | 8.63E-08 | 4.82E-07 | -0.62808 | 7722.07±994.82 | 11934.54±641.81 | urine |
| 2-Benzoxazolol | 153.0658 | 1.016383 | pos | HMDB0032931 | C_7_H_5_NO_2_ | 2.11325 | 4.08E-06 | 1.28E-05 | -0.95744 | 4600.55±720.1 | 8933.62±1523.77 | urine |
| Methyl phenyl disulfide | 139.0035 | 12.8384 | pos | HMDB0040939 | C_7_H_8_S_2_ | 2.08306 | 4.46E-07 | 1.87E-06 | -0.73219 | 6160.66±820.94 | 10233.84±1020.51 | urine |
| Lansoprazole | 350.0566 | 1.71265 | neg | HMDB0005008 | C_16_H_14_F_3_N_3_O_2_S | 2.07869 | 2.01E-11 | 1.29E-09 | -6.29154 | 50.02±27.55 | 3918.37±572.32 | urine |
| Benzoic acid | 105.0338 | 3.187733 | pos | HMDB0001870 | C_7_H_6_O_2_ | 2.05793 | 8.52E-10 | 1.52E-08 | -0.94607 | 4181.24±202.78 | 8055.65±732.09 | urine |
| 3-Mercaptolactate-cysteine disulfide | 286.0059 | 2.471217 | neg | HMDB0006512 | C_6_H_11_NO_5_S_2_ | 2.03448 | 4.03E-08 | 2.65E-07 | -1.7048 | 1770.6±365.29 | 5771.87±992.86 | urine |
| 7,11-Bisdeacetylvaltrate 7-(3-methylpentanoate) 11-(3-hydroxy-3-methylbutanoate) | 539.2488 | 3.903217 | neg | HMDB0033655 | C_26_H_38_O_9_ | 2.01789 | 1.43E-14 | 1.06E-11 | -6.76631 | 33.8±39.57 | 3678.98±315.61 | urine |
| Acitretin Ro 23-4750 | 343.1912 | 6.390483 | pos | 626 | C_21_H_26_O_4_ | 1.96067 | 6.82E-09 | 6.99E-08 | -28.8314 | 0±0 | 3549.36±816.34 | urine |
| 1alpha-hydroxy-22-[3-(1-hydroxy-1-methylethyl)phenyl]-23,24,25,26,27-pentanorvitamin D3 / 1alpha-hydroxy-22-[3-(1-hydroxy-1-methylethyl)phenyl]-23,24,25,26,27-pentanorcholecalciferol | 482.3638 | 12.39528 | pos | LMST03020479 | C_31_H_44_O_3_ | 1.95315 | 5.71E-05 | 0.000141 | 2.14147 | 5096.61±1817.43 | 1155.14±743.78 | urine |
| 38:5(23Z,26Z,29Z,32Z,35Z) | 537.5054 | 14.27057 | pos | LMFA01030837 | C_38_H_66_O_2_ | 1.92319 | 2.27E-06 | 7.67E-06 | -0.77751 | 4964.28±738.28 | 8509.61±1081.82 | urine |
| Niazirin | 297.1456 | 1.606567 | pos | HMDB0032807 | C_14_H_17_NO_5_ | 1.91655 | 0.000953 | 0.001781 | -0.6566 | 7894.21±1257.42 | 12444.16±2822.11 | urine |
| erythro-7,9-Tetratriacontanediol | 549.5018 | 14.609 | pos | HMDB0031281 | C_34_H_70_O_2_ | 1.89685 | 2.82E-06 | 9.14E-06 | -0.99573 | 3479.68±436.55 | 6938.79±1226.6 | urine |
| 4-Hydroxystyrene | 103.0546 | 5.86395 | pos | HMDB0004072 | C_8_H_8_O | 1.87961 | 9.06E-07 | 3.47E-06 | 0.767497 | 8156.8±594.15 | 4791.6±982.9 | urine |
| Prostaglandin G1 | 415.2343 | 9.465167 | neg | HMDB0013039 | C_20_H_34_O_6_ | 1.83089 | 7.02E-07 | 2.78E-06 | 8.186233 | 3276.52±1090.42 | 11.25±21.55 | urine |
| Epiacorone | 219.1749 | 9.550733 | pos | HMDB0031348 | C_15_H_24_O_2_ | 1.76646 | 3.18E-07 | 1.41E-06 | -0.61153 | 5526.79±755.24 | 8444.27±511.48 | urine |
| 1-Nitroheptane | 185.0818 | 12.8384 | pos | HMDB0013811 | C_7_H_16_NO_2_+ | 1.75105 | 7.43E-07 | 2.91E-06 | -0.71417 | 4512.12±556.82 | 7402.29±794.18 | urine |
| Propionylcarnitine | 200.1289 | 5.695567 | pos | HMDB0000824 | C_10_H_19_NO_4_ | 1.74305 | 3.98E-10 | 9.42E-09 | 4.304304 | 2881.75±503.06 | 145.86±58.26 | urine |
| Gibberellin A74 | 345.1725 | 6.22115 | neg | HMDB0038746 | C_20_H_28_O_6_ | 1.73831 | 6.84E-10 | 1.33E-08 | -4.73839 | 108.88±37.55 | 2906.3±538.12 | urine |
| Hexanoylglycine | 385.1666 | 4.747417 | pos | 5669 | C_8_H_15_NO_3_ | 1.68536 | 0.025054 | 0.035455 | 5.508839 | 4440.66±4895.59 | 97.53±123.49 | urine |
| Pentadecanol | 495.4521 | 13.97625 | pos | HMDB0013299 | C_15_H_32_O | 1.61889 | 1.99E-06 | 6.83E-06 | -0.9275 | 2787.53±417.15 | 5301.8±817.75 | urine |
| Butenylcarnitine | 252.1217 | 4.830833 | pos | LMFA07070053 | C_11_H_19_NO_4_ | 1.6115 | 4.26E-08 | 2.76E-07 | 5.366697 | 2507.92±647.73 | 60.78±52.79 | urine |
| 1-docosene | 326.3795 | 12.20573 | pos | HMDB0062602 | C_22_H_44_ | 1.61115 | 0.000366 | 0.000743 | 2.883329 | 3466.45±1776.52 | 469.8±385.46 | urine |
| 11b,17a,21-Trihydroxypreg-nenolone | 365.2329 | 6.137017 | pos | HMDB0006760 | C_21_H_32_O_5_ | 1.60141 | 5.47E-08 | 3.43E-07 | -6.85078 | 20.66±16.17 | 2384.64±640.33 | urine |
| TG(12:0/i-18:0/14:0) | 768.7079 | 14.58777 | pos | HMDB0095830 | C_47_H_90_O_6_ | 1.57593 | 0.012365 | 0.018658 | -0.98857 | 3858.1±3189.34 | 7655.29±1958.85 | urine |
| 36:6(5Z,9Z,12Z,15Z,18Z,21Z)(34Me[S]) | 521.4723 | 14.52393 | pos | LMFA01020341 | C_37_H_62_O_2_ | 1.52092 | 5.57E-06 | 1.69E-05 | -1.511 | 1226.2±376.56 | 3494.75±825.28 | urine |
| 12-Oxo-20-carboxy-leukotriene B4 | 347.1862 | 6.222 | pos | HMDB0012550 | C_20_H_28_O_6_ | 1.50851 | 3.47E-11 | 1.69E-09 | -28.0582 | 0±0 | 2076.78±320.29 | urine |
| N1-(5-Phospho-a-D-ribosyl)-5,6-dimethylbenzimidazole | 357.0843 | 1.753567 | neg | HMDB0003882 | C_14_H_19_N_2_O_7_P | 1.50295 | 0.000153 | 0.00034 | -0.95588 | 2546.42±306.7 | 4939.47±1283.44 | urine |
| 5-hydroxy-1H-indole-3-carboxylic acid | 160.0394 | 1.5019 | pos | HMDB0134931 | C_9_H_7_NO_3_ | 1.49176 | 9.57E-09 | 8.96E-08 | 4.840638 | 2150.55±489.2 | 75.05±29.38 | urine |
| 3,6-dihydroxy-2-phenyl-4H-chromen-4-one | 255.0658 | 7.31755 | pos | HMDB0134546 | C_15_H_10_O_4_ | 1.4909 | 0.00443 | 0.007306 | -0.93025 | 3137.13±353.65 | 5978.16±2346.29 | urine |
| Cholesterol sulfate | 465.3039 | 14.50025 | neg | HMDB0000653 | C_27_H_46_O_4_S | 1.47101 | 0.001389 | 0.002507 | -0.68248 | 4215.86±942.82 | 6766.04±1551.73 | urine |
| Pteroside Z | 377.1967 | 5.92605 | pos | HMDB0032587 | C_21_H_30_O_7_ | 1.44898 | 1.38E-06 | 4.98E-06 | -6.84095 | 17.94±16.42 | 2056.15±721.06 | urine |
| Perlolyrine | 247.0877 | 3.2924 | pos | HMDB0030327 | C_16_H_12_N_2_O_2_ | 1.39351 | 7.56E-08 | 4.38E-07 | 2.41778 | 2278.3±448.42 | 426.37±253.18 | urine |
| Methylscopolamine | 363.1676 | 3.8607 | neg | HMDB0014605 | C_18_H_24_NO_4_+ | 1.38721 | 1.44E-05 | 4.00E-05 | 0.980501 | 3857.99±305.17 | 1955.24±772.16 | urine |
| Kynurenic acid | 190.0502 | 2.597567 | pos | 5683 | C_10_H_7_NO_3_ | 1.38394 | 2.09E-06 | 7.11E-06 | 0.977707 | 3701.11±470.74 | 1879.37±473.87 | urine |
| 5,7-Megastigmadien-9-ol glucoside | 401.2171 | 8.263717 | neg | HMDB0041044 | C_19_H_32_O_6_ | 1.37948 | 6.04E-08 | 3.67E-07 | 3.880185 | 1912.04±467.35 | 129.85±135.9 | urine |
| 2-Hydroxyacorenone | 219.1748 | 10.39427 | pos | HMDB0030916 | C_15_H_24_O_2_ | 1.37815 | 1.88E-07 | 9.21E-07 | -0.58759 | 3533.04±409.31 | 5309.21±339.37 | urine |
| Chlorpropamide | 275.0261 | 3.187283 | neg | HMDB0014810 | C_10_H_13_ClN_2_O_3_S | 1.37242 | 7.21E-06 | 2.14E-05 | -0.69414 | 2962.71±183.41 | 4793.43±726.62 | urine |
| 1-hydroxyoct-2-enoylglycine | 216.1234 | 4.219417 | pos | HMDB0094732 | C_10_H_17_NO_4_ | 1.35523 | 1.45E-11 | 1.13E-09 | 2.457635 | 2009.32±100.3 | 365.79±215.47 | urine |
| Nifursol | 410.0213 | 3.166017 | neg | HMDB0031764 | C_12_H_7_N_5_O_9_ | 1.34002 | 3.82E-11 | 1.80E-09 | -3.26717 | 185.01±148.88 | 1781.19±198.29 | urine |
| Cichorine | 425.1036 | 3.8401 | pos | 68402 | C_10_H_11_NO_3_ | 1.31992 | 0.02676 | 0.037617 | 2.404547 | 3298.11±3052.87 | 622.91±177.93 | urine |
| Platydesminium | 319.142 | 3.608967 | neg | HMDB0030342 | C_16_H_20_NO_3_+ | 1.31756 | 3.12E-07 | 1.39E-06 | 2.802972 | 1957.23±501.19 | 280.45±150.68 | urine |
| Tetranor-5-NO2-CLA | 252.1599 | 6.558883 | pos | LMFA01120010 | C_14_H_23_NO_4_ | 1.31687 | 1.02E-05 | 2.93E-05 | -1.0677 | 1597.87±290.56 | 3349.27±680.65 | urine |
| 5-hydroxyhexanoylglycine | 172.0974 | 1.37445 | pos | HMDB0094722 | C_8_H_15_NO_4_ | 1.31657 | 9.31E-09 | 8.75E-08 | 1.782469 | 2226.21±224.05 | 647.13±297.04 | urine |
| 15-Keto-13,14-dihydroprostaglandin A2 | 379.2138 | 6.894517 | neg | HMDB0001244 | C_20_H_30_O_4_ | 1.30101 | 2.33E-11 | 1.38E-09 | -27.6083 | 0±0 | 1520.47±227.7 | urine |
| Terbutaline | 226.1442 | 4.07225 | pos | 2762 | C_12_H_19_NO_3_ | 1.28918 | 1.95E-08 | 1.50E-07 | 2.560586 | 1863.92±381.25 | 315.95±63.03 | urine |
| 4,7,10,13,16-Docosapentaynoic acid | 301.1598 | 10.98238 | neg | LMFA01030678 | C_22_H_24_O_2_ | 1.28652 | 5.15E-11 | 2.27E-09 | 27.5961 | 1507.64±239.43 | 0±0 | urine |
| Medicoside G | 827.4463 | 12.8384 | pos | HMDB0038622 | C_42_H_66_O_16_ | 1.28318 | 1.01E-06 | 3.83E-06 | -0.67525 | 2629.19±383.72 | 4198.47±380.7 | urine |
| N-Methylmescaline | 226.144 | 4.325717 | pos | 3364 | C_12_H_19_NO_3_ | 1.27524 | 6.45E-08 | 3.87E-07 | 2.305949 | 1915.85±410.6 | 387.44±86.51 | urine |
| Pipercitine | 332.3322 | 11.99477 | pos | HMDB0039937 | C_23_H_43_NO | 1.27487 | 0.001064 | 0.001973 | 1.353508 | 3273.7±1352.01 | 1281.13±231.58 | urine |
| (E)-3,7-Dimethyl-1,5,7-octatrien-3-ol | 153.1276 | 9.33985 | pos | HMDB0032242 | C_10_H_16_O | 1.27303 | 1.65E-07 | 8.31E-07 | 1.034551 | 2959.96±310.52 | 1444.95±324.13 | urine |
| 1-Methylinosine | 263.079 | 1.6277 | neg | HMDB0002721 | C_11_H_14_N_4_O_5_ | 1.26093 | 2.33E-11 | 1.38E-09 | 1.840223 | 1975.21±143 | 551.63±158.05 | urine |
| Boldione | 307.1699 | 6.390483 | pos | HMDB0003422 | C_19_H_24_O_2_ | 1.23992 | 1.16E-07 | 6.14E-07 | -5.61152 | 30.55±29 | 1493.81±420.13 | urine |
| Aldosterone | 361.2017 | 9.192683 | pos | HMDB0000037 | C_21_H_28_O_5_ | 1.23844 | 5.71E-09 | 6.10E-08 | -27.504 | 0±0 | 1414.38±320.85 | urine |
| 4-Hydroxycinnamoylagmatine | 259.1549 | 12.8384 | pos | HMDB0033460 | C_14_H_20_N_4_O_2_ | 1.2232 | 4.22E-07 | 1.79E-06 | -0.694 | 2267.82±264.03 | 3668.79±362.41 | urine |
| N-lactoyl-Methionine | 204.07 | 2.07085 | pos | HMDB0062182 | C_8_H_15_NO_4_S | 1.21989 | 3.62E-08 | 2.41E-07 | 4.695591 | 1436.71±360.73 | 55.44±32.14 | urine |
| (2R,2'S)-Isobuteine | 206.0495 | 2.471217 | neg | HMDB0030411 | C_7_H_13_NO_4_S | 1.21244 | 2.18E-08 | 1.64E-07 | -1.71764 | 614.25±107.34 | 2020.25±337.47 | urine |
| 5,6-Epoxy-8,11,14-eicosatrienoic acid | 365.2337 | 8.517083 | neg | HMDB0002190 | C_20_H_32_O_3_ | 1.20518 | 0.001338 | 0.002423 | -7.21349 | 12.47±28.16 | 1851.16±1302.61 | urine |
| Leukoaminochrome | 134.0604 | 3.166483 | pos | HMDB0012992 | C_8_H_9_NO_2_ | 1.20183 | 4.61E-09 | 5.19E-08 | 1.158277 | 2457.45±204.85 | 1101.05±222.79 | urine |
| Kaempferol 3-rhamnosyl-(1->2)-[xylosyl-(1->3)-rhamnosyl-(1->6)-galactoside] | 890.2933 | 9.045533 | pos | LMPK12111932 | C_38_H_48_O_23_ | 1.1921 | 3.42E-05 | 8.79E-05 | 9.675418 | 1476.89±698.69 | 1.81±5.11 | urine |
| Cer(d14:2(4E,6E)/20:1(11Z)) | 551.5142 | 14.4602 | pos | LMSP02010055 | C_34_H_63_NO_3_ | 1.17008 | 7.82E-06 | 2.30E-05 | -0.80828 | 1791.86±347.62 | 3137.78±432.68 | urine |
| Canavanine | 221.0887 | 14.66865 | neg | HMDB0002706 | C_5_H_12_N_4_O_3_ | 1.15658 | 1.94E-07 | 9.44E-07 | -0.8731 | 1487.74±257.95 | 2724.93±267.19 | urine |
| L-DOPA 3'-glucoside | 382.1102 | 2.386583 | pos | HMDB0029452 | C_15_H_21_NO_9_ | 1.15496 | 2.18E-06 | 7.36E-06 | 6.44607 | 1316.49±478.11 | 15.1±27.96 | urine |
| 7-Isothiocyanato-1-heptene | 311.1608 | 2.092183 | pos | HMDB0038434 | C_8_H_13_NS | 1.15395 | 4.75E-09 | 5.32E-08 | 0.991375 | 2439.62±205.76 | 1227.12±176.59 | urine |
| Dimethylarsinous acid | 102.9537 | 14.90075 | neg | HMDB0012216 | C_2_H_7_AsO | 1.14837 | 8.36E-08 | 4.70E-07 | -1.02972 | 1163.05±146.84 | 2374.51±306.22 | urine |
| [2-hydroxy-1-(7-hydroxy-1-oxo-1H-isochromen-3-yl)butoxy]sulfonic acid | 329.0326 | 2.428717 | neg | HMDB0130071 | C_13_H_14_O_8_S | 1.14108 | 3.49E-11 | 1.69E-09 | 27.23565 | 1174.34±181.18 | 0±0 | urine |
| (E)-2-octenal | 109.1013 | 4.515267 | pos | HMDB0013809 | C_8_H_14_O | 1.1392 | 1.58E-08 | 1.29E-07 | 2.87719 | 1395.28±291.82 | 189.91±49.24 | urine |
| docebenone | 309.1857 | 9.045533 | pos | LMFA05000652 | C_21_H_26_O_3_ | 1.09954 | 1.33E-05 | 3.72E-05 | -27.2973 | 0±0 | 1225.57±530.77 | urine |
| 6-hydroxyoct-6-enoylglycine | 216.1238 | 2.934367 | pos | HMDB0094771 | C_10_H_17_NO_4_ | 1.09852 | 8.09E-09 | 7.92E-08 | 3.196123 | 1232.78±222.35 | 134.51±126.77 | urine |
| Hydrocortisone succinate | 507.2226 | 8.8538 | neg | LMST02030126 | C_25_H_34_O_8_ | 1.09681 | 0.000607 | 0.001178 | -27.4162 | 0±0 | 1330.93±855.98 | urine |
| 4-oxo-Retinoic acid | 359.1878 | 8.6217 | neg | HMDB0006285 | C_20_H_26_O_3_ | 1.08945 | 3.42E-07 | 1.49E-06 | -9.15627 | 2.02±5.72 | 1153.54±362.15 | urine |
| Ethyl trans-p-methoxycinnamate | 224.1283 | 10.584 | pos | HMDB0030762 | C_12_H_14_O_3_ | 1.08597 | 1.78E-06 | 6.19E-06 | -0.67855 | 1865.01±288.78 | 2985.01±283.88 | urine |
| 11-dehydro-2,3-dinor-TXB2 | 385.1876 | 5.6944 | neg | LMFA03030013 | C_18_H_28_O_6_ | 1.08228 | 0.00169 | 0.003001 | -2.13875 | 420.4±50.44 | 1851.35±1043.84 | urine |
| PC(O-15:0/0:0) | 466.33 | 12.37183 | neg | LMGP01060009 | C_23_H_50_NO_6_P | 1.08206 | 3.42E-05 | 8.79E-05 | 1.784254 | 1698.07±469.08 | 492.99±325.32 | urine |
| NTP | 432.9725 | 1.50165 | neg | HMDB0060500 | C_6_H_15_O_13_P_3_ | 1.07957 | 2.30E-07 | 1.08E-06 | 27.1721 | 1123.73±342.06 | 0±0 | urine |
| Thonzylamine | 285.173 | 3.671017 | neg | HMDB0240222 | C_16_H_22_N_4_O | 1.06943 | 1.07E-08 | 9.75E-08 | -1.92676 | 381.92±66.62 | 1452.05±246.05 | urine |
| LysoPE(0:0/24:1(15Z)) | 586.384 | 7.9289 | pos | HMDB0011498 | C_29_H_58_NO_7_P | 1.05836 | 0.000482 | 0.000956 | 27.35051 | 1271.65±796.13 | 0±0 | urine |
| 1-(1,2,3,4,5-Pentahydroxypent-1-yl)-1,2,3,4-tetrahydro-beta-carboline-3-carboxylate | 367.1515 | 1.395683 | pos | HMDB0012492 | C_17_H_22_N_2_O_7_ | 1.05024 | 3.34E-06 | 1.07E-05 | 1.256512 | 1849.99±196.99 | 774.32±360.71 | urine |
| Kaempferol 3-sophoroside 7-glucuronide | 767.1714 | 9.001033 | neg | HMDB0038769 | C_33_H_38_O_22_ | 1.04518 | 2.21E-08 | 1.65E-07 | 4.54668 | 1074.84±252.79 | 45.99±58.15 | urine |
| Canavaninosuccinate | 273.0833 | 6.4531 | neg | HMDB0012197 | C_9_H_16_N_4_O_7_ | 1.04346 | 0.011577 | 0.017568 | 5.05736 | 1635.12±1545.25 | 49.11±18.35 | urine |
| 1,1'-Bis(2-hydroxy-3-methylcarbazole) | 437.1497 | 5.6732 | neg | HMDB0040776 | C_26_H_20_N_2_O_2_ | 1.03807 | 0.017099 | 0.025087 | 27.71254 | 1634.37±1709.14 | 0±0 | urine |
| 17a-Ethynylestradiol | 297.1844 | 6.411733 | pos | HMDB0001926 | C_20_H_24_O_2_ | 1.03537 | 3.44E-07 | 1.49E-06 | -27.0401 | 0±0 | 1025.5±322.72 | urine |
| N-Propionylmethionine | 204.0707 | 5.841633 | neg | HMDB0094704 | C_8_H_15_NO_3_S | 1.03315 | 0.000563 | 0.001099 | 0.446305 | 4502.87±106.49 | 3304.75±756.3 | urine |
| Lactyltrimethylammonium betaine | 188.9903 | 1.71265 | neg | HMDB0032684 | C_6_H_13_AsO_3_ | 1.03278 | 6.92E-05 | 0.000167 | -1.83941 | 437.29±31.2 | 1564.89±571.88 | urine |
| 2,3-Dinor-6-keto-prostaglandin F1 a | 387.2033 | 5.777817 | neg | HMDB0002277 | C_18_H_30_O_6_ | 1.0301 | 1.06E-05 | 3.04E-05 | 0.472405 | 3956.69±142.11 | 2851.83±446.6 | urine |
| GlcCer(d18:0/16:0) | 684.5792 | 14.10217 | pos | LMSP0501AA04 | C_40_H_79_NO_8_ | 1.02374 | 0.000229 | 0.000486 | -0.69521 | 1861.99±364.33 | 3014.8±554.95 | urine |
| Ibuprofen | 413.2688 | 14.73492 | pos | HMDB0001925 | C_13_H_18_O_2_ | 1.01348 | 0.000123 | 0.000279 | -0.43986 | 3244.07±438.99 | 4400.5±442.15 | urine |
| 3-(Methylthio)propyl acetate | 193.0544 | 3.208483 | neg | HMDB0031717 | C_6_H_12_O_2_S | 1.00596 | 0.007527 | 0.011885 | -0.65497 | 2363.95±354.88 | 3722.23±1179.09 | urine |

| Sample | clean_reads | clean_bases | Read length | Q30 | GC |
| --- | --- | --- | --- | --- | --- |
| Sample_Con1 | 48.31 million | 7.04G | 150 | 94.52% | 48.48% |
| Sample_Con2 | 48.37 million | 7.08G | 150 | 94.80% | 48.65% |
| Sample_Con3 | 48.10 million | 7.01G | 150 | 93.78% | 48.65% |
| Sample_MDL1 | 48.30 million | 7.07G | 150 | 94.51% | 48.70% |
| Sample_MDL2 | 48.34 million | 7.08G | 150 | 94.70% | 48.10% |
| Sample_MDL3 | 48.33 million | 7.08G | 150 | 94.50% | 48.06% |
| Sample_MH1 | 48.35 million | 7.08G | 150 | 94.51% | 48.21% |
| Sample_MH2 | 48.28 million | 7.06G | 150 | 94.34% | 48.84% |
| Sample_MH3 | 48.36 million | 7.08G | 150 | 94.59% | 48.13% |
| Sample_TJH1 | 48.30 million | 7.07G | 150 | 94.68% | 47.54% |
| Sample_TJH2 | 48.20 million | 7.05G | 150 | 94.19% | 47.93% |
| Sample_TJH3 | 48.23 million | 7.07G | 150 | 94.21% | 47.90% |
| Sample_VGH1 | 48.24 million | 7.05G | 150 | 94.37% | 48.01% |
| Sample_VGH2 | 48.36 million | 7.08G | 150 | 94.57% | 47.95% |
| Sample_VGH3 | 48.30 million | 7.07G | 150 | 94.43% | 47.79% |

**Table S6** The results of sequencing data quality preprocess

**Table S7** Power value filtering

| Power | SFT. R. sq | slope | truncated. R. sq | mean. k. | median. k. | max. k. |
| --- | --- | --- | --- | --- | --- | --- |
| 1 | 0.268644 | 5.560381 | 0.905692 | 7975.254 | 8027.195 | 9104.515 |
| 2 | 0.203126 | 2.431627 | 0.899424 | 4707.539 | 4658.277 | 6078.365 |
| 3 | 0.082357 | 0.921785 | 0.86064 | 3030.622 | 2965.797 | 4525.659 |
| 4 | 0.005154 | 0.153243 | 0.827029 | 2077.055 | 2002.637 | 3590.161 |
| 5 | 0.049326 | -0.34953 | 0.824137 | 1492.63 | 1406.56 | 2963.213 |
| 6 | 0.256265 | -0.70402 | 0.8654 | 1113.246 | 1013.91 | 2528.634 |
| 7 | 0.491221 | -0.9511 | 0.907855 | 855.4546 | 752.4673 | 2200.667 |
| 8 | 0.644063 | -1.11458 | 0.92394 | 673.6452 | 569.0261 | 1944.288 |
| 9 | 0.725696 | -1.22784 | 0.929873 | 541.4004 | 438.0803 | 1738.241 |
| 10 | 0.77749 | -1.3129 | 0.932151 | 442.6704 | 343.4762 | 1568.912 |
| 12 | 0.834248 | -1.41223 | 0.936732 | 308.6611 | 219.9124 | 1306.784 |
| 14 | 0.867076 | -1.46756 | 0.944746 | 225.0057 | 146.3119 | 1113.016 |
| 16 | 0.870624 | -1.51577 | 0.936544 | 169.7719 | 100.4222 | 963.8023 |
| 18 | 0.877108 | -1.53779 | 0.936819 | 131.6555 | 70.4432 | 845.3332 |
| 20 | 0.876885 | -1.55039 | 0.933543 | 104.397 | 50.61814 | 749.0235 |
| 22 | 0.879812 | -1.56212 | 0.934817 | 84.32388 | 37.09593 | 669.2421 |
| 24 | 0.880423 | -1.56341 | 0.935138 | 69.1758 | 27.55512 | 602.1326 |
| 26 | 0.881377 | -1.57033 | 0.936725 | 57.50499 | 20.80688 | 544.96 |
| 28 | 0.878268 | -1.57583 | 0.933301 | 48.3521 | 15.93193 | 495.7273 |


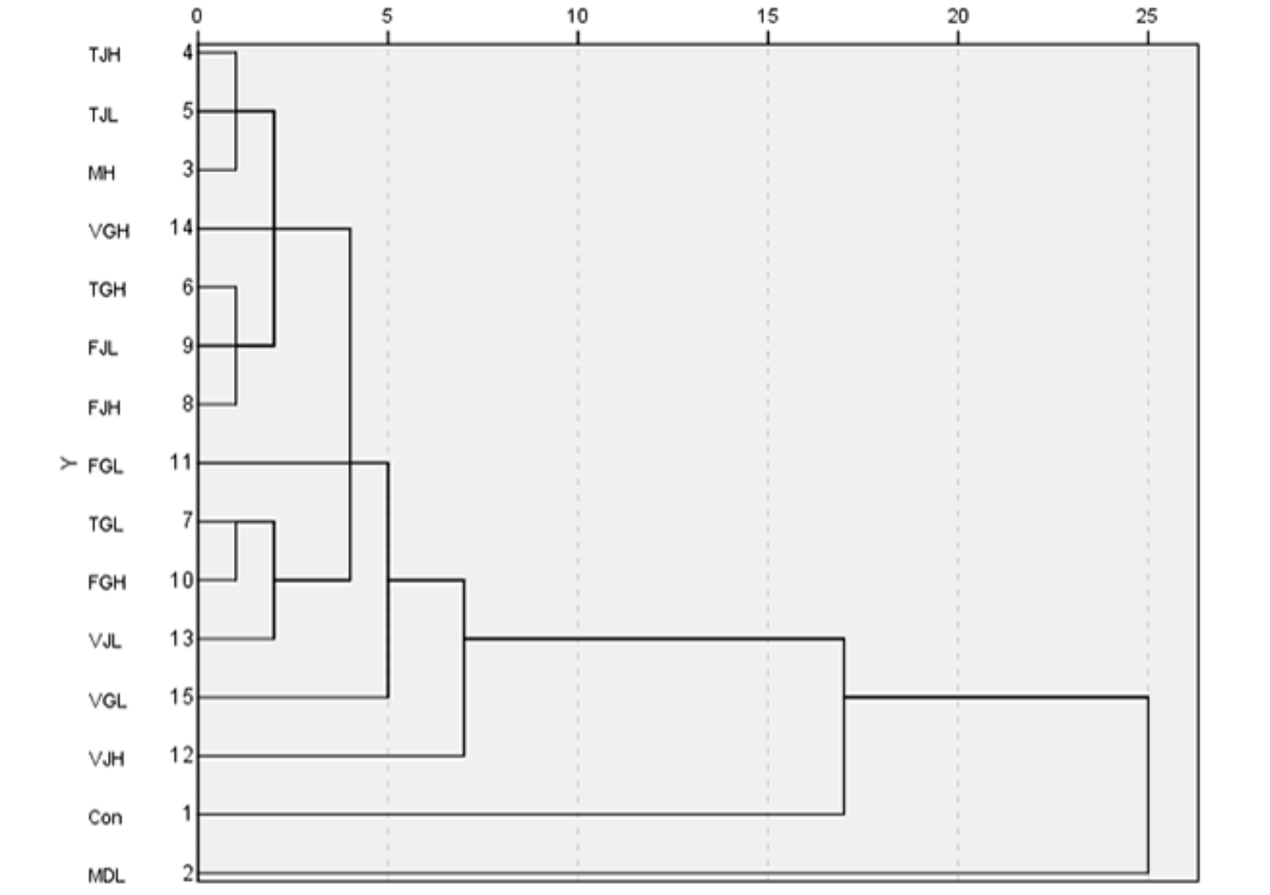


**Figure S1** Cluster analysis of biochemical indicators in db/db mice


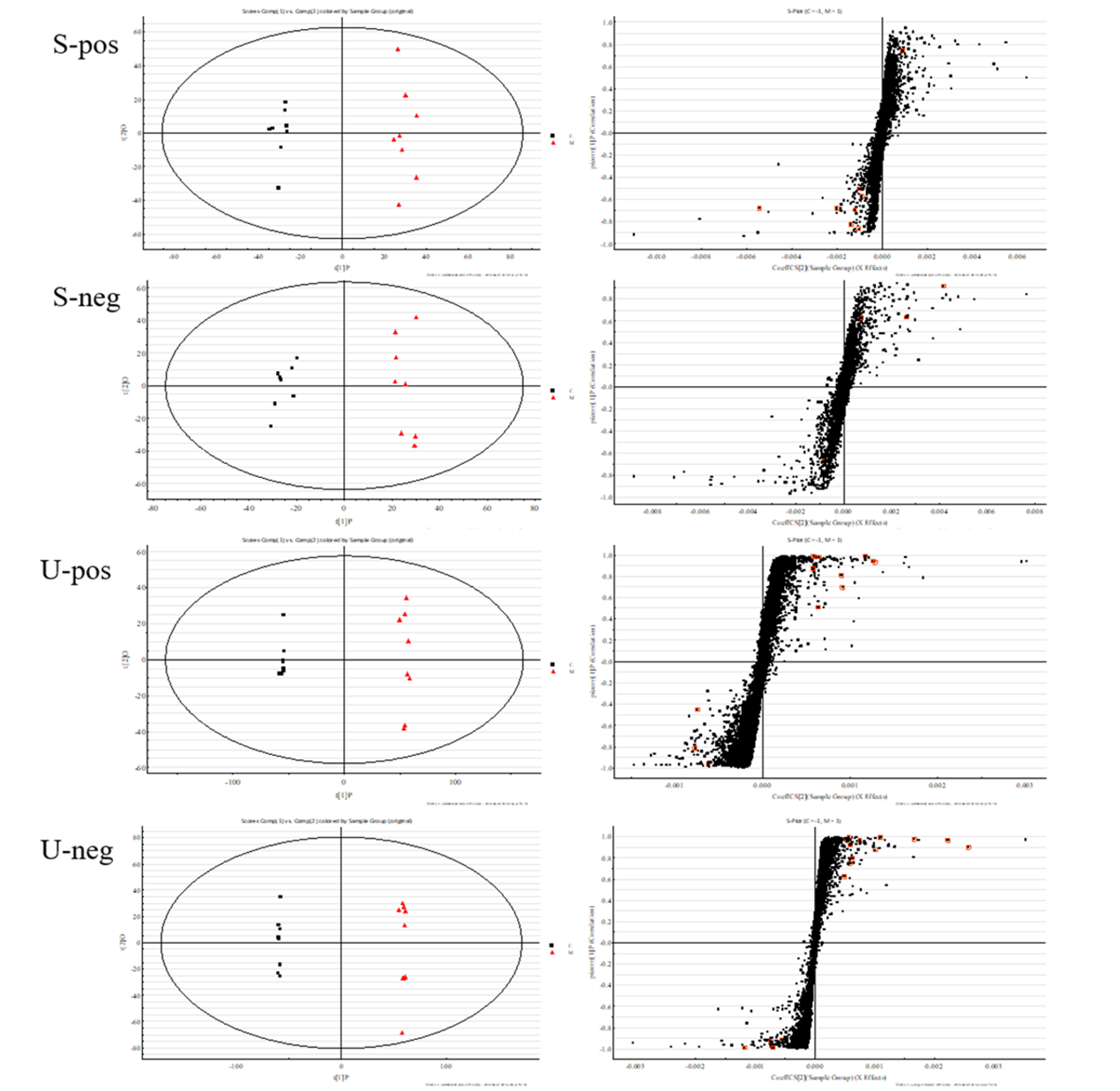


**Figure S2** OPLS-DA scores plots, S-plot of OPLS-DA for serum (S) and urine (U) samples of model group (red) and control group (black) in positive (pos) and negative (neg) ion mode.


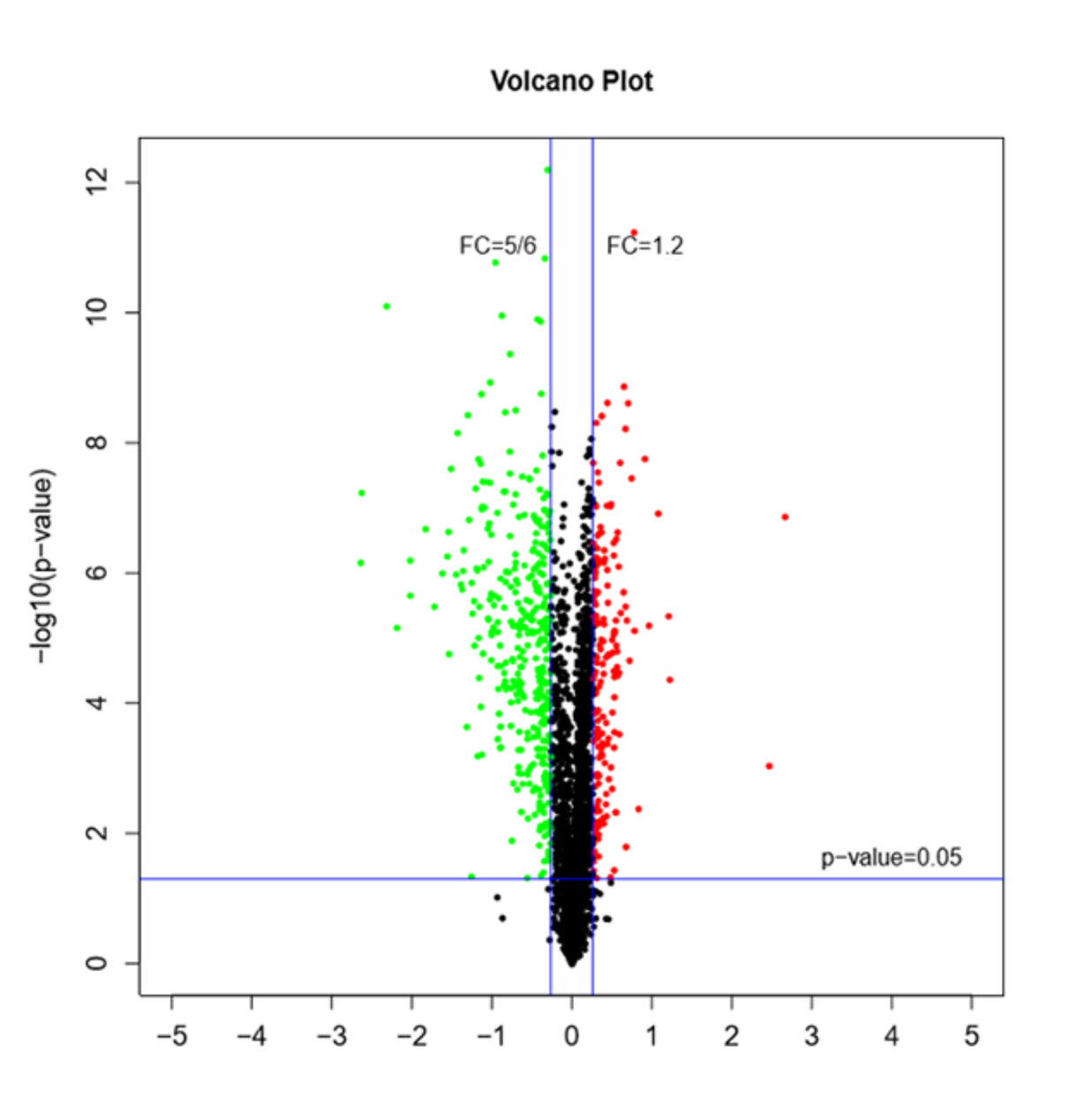


**Figure S3** Volcano maps (Model vs Control) (green dots: down-regulated; red dots: up-regulated; black dots: non-significant)
